# Stage-Specific Changes in *Plasmodium* Metabolism Required for Differentiation and Adaptation to Different Host and Vector Environments

**DOI:** 10.1371/journal.ppat.1006094

**Published:** 2016-12-27

**Authors:** Anubhav Srivastava, Nisha Philip, Katie R. Hughes, Konstantina Georgiou, James I. MacRae, Michael P. Barrett, Darren J. Creek, Malcolm J. McConville, Andrew P. Waters

**Affiliations:** 1 Wellcome Centre for Molecular Parasitology, Institute of Infection, Immunity & Inflammation, College of Medical, Veterinary and Life Sciences, University of Glasgow, Glasgow, United Kingdom; 2 Department of Biochemistry and Molecular Biology, Bio21 Molecular Science and Biotechnology Institute, University of Melbourne, Parkville, Victoria, Australia; 3 Glasgow Polyomics, Wolfson Wohl Cancer Research Centre, Garscube Campus, Bearsden, University of Glasgow, Glasgow, United Kingdom; 4 Metabolomics Australia, Bio21 Molecular Science and Biotechnology Institute, University of Melbourne, Parkville, Victoria, Australia; Washington University School of Medicine, UNITED STATES

## Abstract

Malaria parasites (*Plasmodium* spp.) encounter markedly different (nutritional) environments during their complex life cycles in the mosquito and human hosts. Adaptation to these different host niches is associated with a dramatic rewiring of metabolism, from a highly glycolytic metabolism in the asexual blood stages to increased dependence on tricarboxylic acid (TCA) metabolism in mosquito stages. Here we have used stable isotope labelling, targeted metabolomics and reverse genetics to map stage-specific changes in *Plasmodium berghei* carbon metabolism and determine the functional significance of these changes on parasite survival in the blood and mosquito stages. We show that glutamine serves as the predominant input into TCA metabolism in both asexual and sexual blood stages and is important for complete male gametogenesis. Glutamine catabolism, as well as key reactions in intermediary metabolism and CoA synthesis are also essential for ookinete to oocyst transition in the mosquito. These data extend our knowledge of *Plasmodium* metabolism and point towards possible targets for transmission-blocking intervention strategies. Furthermore, they highlight significant metabolic differences between *Plasmodium* species which are not easily anticipated based on genomics or transcriptomics studies and underline the importance of integration of metabolomics data with other platforms in order to better inform drug discovery and design.

## Author Summary

Malaria kills almost half a million people worldwide every year and more than two hundred million people are diagnosed with this deadly disease annually. It is caused by the protozoan parasite *Plasmodium* spp., mostly in sub-Saharan Africa and Asia and is transmitted by bites of infected female *Anopheles* mosquitoes. Due to an increase in resistance to existing drugs and lack of an effective vaccine, new intervention strategies which target development of parasite in human host and transmission through the mosquito vector are urgently needed. In this study, we explored the metabolic capacity of different developmental stages of the malaria parasite to determine carbon source utilization in different host niches and whether any stage-specific switches in metabolism could be exploited in new therapies aimed at eradicating malaria. Using stable isotope labelling and metabolomics, we have identified considerable nutritional adaptability of malaria parasites between the mammalian host and the mosquito vector. Gene disruption in the rodent malaria parasite *P*. *berghei* was used to identify the metabolic pathways which are crucial to the survival and development of the parasite. Our data also point at key metabolic differences in different *Plasmodium* species highlighting the importance of integrating metabolomics analyses with molecular tools and identifies possible transmission blocking candidates for malaria intervention.

## Introduction

Malaria remains a major public health problem with more than 214 million new cases each year and 438,000 deaths world-wide. Significant progress has been made in reducing the number of cases of malaria over the last 15 years, largely as a result of the introduction of different control interventions, including artemisinin-based combination therapies [1]. However, increasing resistance to artemisinin threatens to undermine existing malaria control programs [2] and there remains an ongoing need to develop new therapeutics with a particular focus on drugs that target different parasite developmental stages responsible for pathogenesis and transmission via the mosquito vector.

All of the symptoms and pathology associated with malaria are linked to the repeated cycles of infection and lysis of host red blood cells (RBC) completed by asexual blood stages of *Plasmodium*. Glucose consumption by *Plasmodium*-infected RBC increases 10-fold and these stages rely primarily on glycolysis for energy generation [3–7]. Notwithstanding their dependence on glycolysis, asexual blood stages maintain a single, poorly cristate mitochondrion [8] and are dependent on electron transport chain (ETC) activity for the re-oxidation of inner membrane dehydrogenases and pyrimidine biosynthesis [9]. The maintenance of the mitochondrial ETC is sustained in part, by the oxidation of pyruvate (diverted from glycolysis) and the uptake and catabolism of glutamine [7,10,11]. Pyruvate can enter the TCA cycle via two pathways; through anaplerotic reactions involving the CO_2_-fixing enzyme, phosphoenolpyruvate carboxylase (PEPC), or through the activity of a repurposed branched chain α-keto acid dehydrogenase (BCKDH) complex, which substitutes for the activity of the missing mitochondrial pyruvate dehydrogenase in *Plasmodium* and other apicomplexan parasites [12,13]. Despite the essentiality of the mitochondrion, operation of the TCA cycle is not required for intra-erythrocytic growth of *P*. *falciparum* [10,11,14,15].

*Plasmodium* spp. lack key enzymes involved in gluconeogenesis and all developmental stages are predicted to be dependent on the uptake of sugars. However, in contrast to the asexual blood stages, there is increasing evidence that the mosquito-infective stages of *Plasmodium* exhibit an increased dependence on the TCA cycle and mitochondrial metabolism [11,14,16,17]. In particular, transcriptomic and proteomic analyses [5,18–20] suggest that enzymes involved in TCA metabolism are elevated in *Plasmodium* mosquito stages. Specifically, *Plasmodium* gametocytes develop more complex tubular mitochondrial cristae suggestive of increased mitochondrial function [21]. Metabolomic analyses have confirmed increased TCA metabolism in *P*. *falciparum* gametocytes and demonstrated that this is essential for gametocyte maturation [10]. Recent genetic studies have also shown that the TCA cycle is essential for the development of *P*. *falciparum* mosquito stages [11], consistent with earlier work in *P*. *berghei* demonstrating that the TCA cycle and the electron transport chain are required for ookinete development and oocyst formation [14]. Interestingly, levels of sugars and glutamate/glutamine in the mosquito haemolymph are thought to be comparable to the levels in the blood [22–25] suggesting that these stage-specific shifts in parasite metabolism may be driven by factors other than the availability of specific carbon sources in the mosquito niches.

Little is known about the metabolic flexibility of different *Plasmodium* mosquito stages and the extent to which parasite metabolism impacts on the development of these stages in the mosquito vector. In this study we have utilized a combination of metabolomic and reverse genetic approaches to investigate the metabolic changes that occur in key insect stages of the experimentally tractable species *P*. *berghei* and the potential impact of these changes on parasite infection in the mosquito. We find that these stages are highly sensitive to disruptions in multiple pathways in central carbon metabolism including the TCA cycle, the utilisation of glutamine as a carbon source, intermediary carbon metabolism and coenzyme A (CoA) synthesis. The strict dependency of these stages on multiple pathways in carbon metabolism opens new avenues for targeting parasite transmission.

## Results

*P*. *berghei* asexual blood stages, gametocytes and ookinete stages were cultured in minimal medium containing AlbuMAX and metabolically labelled with U-^13^C-glucose or U-^13^C^15^N-glutamine (‘U’ indicates metabolites where all of the indicated atoms are isotopically labelled). The extent to which these stages are dependent on glycolysis and the TCA cycle and related intermediary metabolism was subsequently inferred from analysis of isotopic enrichment of intracellular metabolite pools by gas chromatography-mass spectrometry (GC-MS). *P*. *berghei* mutant lines lacking key enzymes in glucose and glutamine catabolism were also generated to further assess the functional significance of specific metabolic pathways on parasite development and survival. The following sections describe the analysis of each developmental stage.

### *P*. *berghei* asexual blood stages are dependent on glycolysis but also operate a limited TCA cycle

Uninfected RBC (uRBC) and synchronised *P*. *berghei* ring stage infected RBC populations (iRBC, 10% parasitaemia) containing ~35% reticulocytes were generated as described previously [26]. uRBC and iRBC were metabolically labelled with U-^13^C-glucose and U-^13^C^15^N-glutamine and harvested at various time points (0, 6, 12, 18 and 24 h) over the intra-erythrocytic developmental cycle. Asexual parasites from a recently generated gametocyte non-producer line (GNPm9) [27] were used to avoid any low level contamination of RBC asexual stages with gametocytes.

#### ^13^C U-glucose and ^13^C^15^N U-glutamine labelling during asexual growth

**Fig 1:**
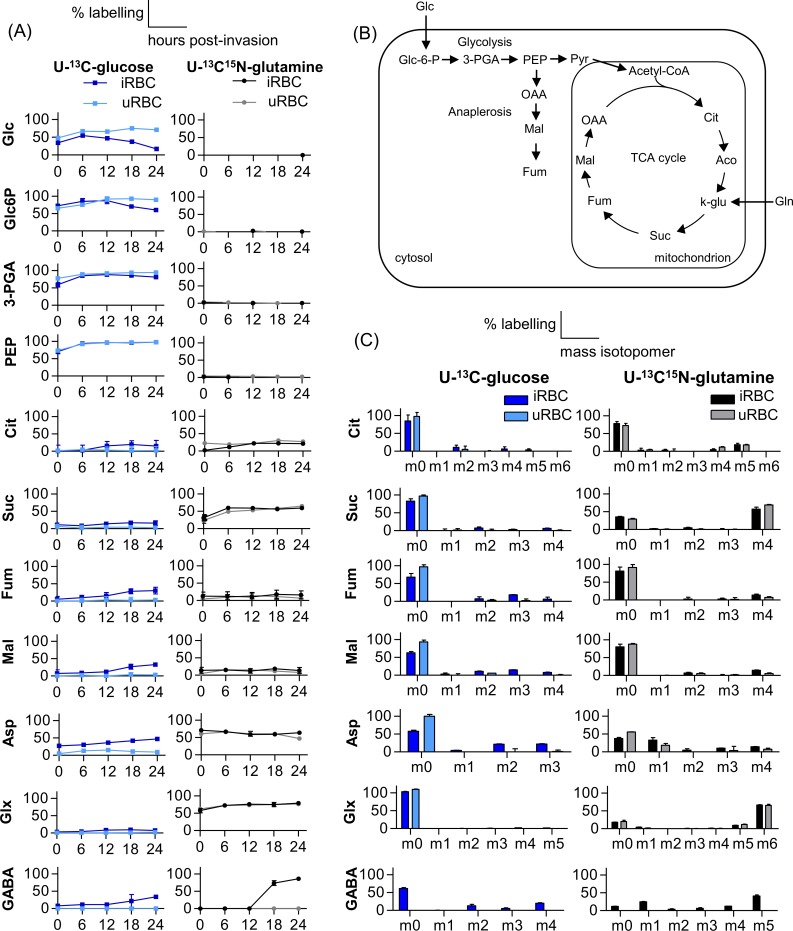
U-^13^C-glucose and U-^13^C^15^N-glutamine labelling of glycolytic and TCA cycle intermediates in *P*. *berghei* asexual stages. (A) *P*. *berghei* infected RBC (iRBC) and uninfected RBC (uRBC) were metabolically labelled with U-^13^C-glucose and U-^13^C^15^N-glutamine for indicated times (0, 6, 12, 18, 24 h post-invasion) and percent labelling of indicated metabolites (mol. % containing one or more ^13^C carbons after correction for natural abundance) determined by GC/MS. (B) Schematic representation of entry of glucose and glutamine as carbon sources and their anticipated fate through glycolysis and TCA cycle. (C) Mass isotopologue distributions of TCA-cycle metabolites at the 24 h time point (schizont stage) in iRBC) and uRBC cultured in the presence of U-^13^C-glucose and U-^13^C^15^N-glutamine. The x-axis indicates the number of ^13^C atoms in each metabolite (the ion used to analyse aspartate contains 3 of the 4 carbons as the 4-carbon fragment was below the limit of quantification). Due to the presence of a labelled nitrogen atom when labelling with U-^13^C^15^N-glutamine, the isotopologue analyses of nitrogen-containing metabolites include an isotope 1 dalton higher than the U-^13^C-glucose equivalent; i.e. aspartate (Asp, +4), glutamine/glutamate (Glx, +6) and ɣ-aminobutyric acid (GABA, +5). Error bars indicate SD of n = 3 biological replicates. Abbreviations used; glucose (Glc), glucose 6-phosphate (Glc-6-P), 3-phosphoglycerate (3-PGA), phosphoenolpyruvate (PEP), citrate (Cit), succinate (Suc), fumarate (Fum), malate (Mal), aspartate (Asp), glutamine/glutamate (Glx) and ɣ-aminobutyric acid (GABA), Gln, glutamine; Co-A, coenzyme-A; Pyr, pyruvate; OAA, oxaloacetate; Aco, aconitate; k-glu, α-ketoglutarate.

Consistent with earlier studies, both uRBC and iRBC populations rapidly incorporated U-^13^C-glucose into glycolytic intermediates such as glucose 6-phosphate (Glc6P), 3-phosphoglycerate (3-PGA) and phosphoenolpyruvate (PEP) (Fig 1A). Interestingly, percent ^13^C-enrichment in both glucose and Glc6P in iRBC decreased in schizont stages possibly reflecting depletion of U-^13^C-glucose from the media due to the highly active glycolytic metabolism in this stage (Fig 1A). The presence of unlabelled glucose in these cells was presumably from pre-existing pools in erythrocytes. Detectable ^13^C labelling of most TCA cycle intermediates including citrate, succinate, fumarate, malate and aspartate (which is derived from oxaloacetate, OAA) were also detected in iRBC, but minimal labelling of these metabolites was observed in uRBC (Fig 1A). Since mature erythrocytes lack a TCA cycle, the residual labelling of these intermediates in uRBC is most likely due to the presence of reticulocytes in these preparations (which typically constitute 35% of these cultures). Operation of a canonical TCA cycle (Fig 1B) in which pyruvate enters the TCA cycle via the BCKDH complex [10,13] was indicated by detailed isotopologue analysis of the TCA cycle metabolites in schizont infected RBC (24 h time point) (Fig 1C). Specifically, detection of +2 and +4 isotopologues of citrate indicated entry of pyruvate-derived acetyl-CoA into the TCA cycle, while the presence of +3 labelled carbons in fumarate, malate and aspartate (Fig 1C) was consistent with the conversion of PEP to C4 carboxylic acids via the activity of PEP carboxylase (PEPC) (see S1 Fig for details of isotopologue analysis).

The low level of U-^13^C-glucose labelling of TCA cycle intermediates in *P*. *berghei* asexual blood stages (< 20%) indicated that these stages may co-utilize glutamine, which also feeds directly into the TCA cycle (Fig 1B). Consistent with this notion, labelling of uRBC and iRBC with U-^13^C^15^N-glutamine resulted in significant labelling of all TCA intermediates (Fig 1A). Significant differences were observed in the level of labelling of individual TCA cycle intermediates (elevated in succinate, aspartate, and citrate compared to fumarate and malate) reflecting differences in the relative size and extent to which cytoplasmic and mitochondrial pools of these metabolites are connected to glycolysis and/or mitochondrial metabolism. Isotopologue analysis of U-^13^C^15^N-glutamine labelling in asexual *P*. *berghei* 24 h schizonts (and uRBC containing 35% reticulocytes) showed that all TCA cycle metabolites had the expected +2 and +4 labelling (Fig 1C) which was higher in fumarate and malate in iRBC. The +4 labelling was higher than that seen in U-^13^C-glucose labelled cells, indicating that glutamine is the primary carbon source used to drive the TCA cycle. The +4 label was maximal in intermediates such as succinate, immediately downstream of α-ketoglutarate, the point of entry of labelled glutamine carbon skeletons. Significantly, we also observed +5 citrate isotopologues in both uRBC and iRBC indicating the background levels of reductive catabolism of exogenous glutamine by host cells (presumably the reticulocyte population).

We have previously shown that *P*. *falciparum*, and other apicomplexan parasites, can convert glutamate to ɣ-aminobutyric acid (GABA) [10,28]. In *Toxoplasma gondii*, GABA can be further catabolized to succinyl-CoA which can enter the TCA cycle. However, *P*. *falciparum* and *P*. *berghei* lack the enzymes needed to generate succinyl-CoA and may primarily utilize GABA to maintain transamination reactions in the mitochondrion and/or cytoplasm [10]. U-^13^C^15^N-glutamine labelling of *P*. *berghei* parasites showed that during asexual development, only trophozoites and mature schizonts actively produce GABA (Fig 1A) and isotopologue analysis of these cells also showed labelling of GABA at 24 h schizont stage. No GABA was detected in uRBC (Fig 1A and 1C), confirming that the production of GABA was parasite-specific.

#### Genetic dissection of central carbon metabolism in asexual blood stages

**Fig 2:**
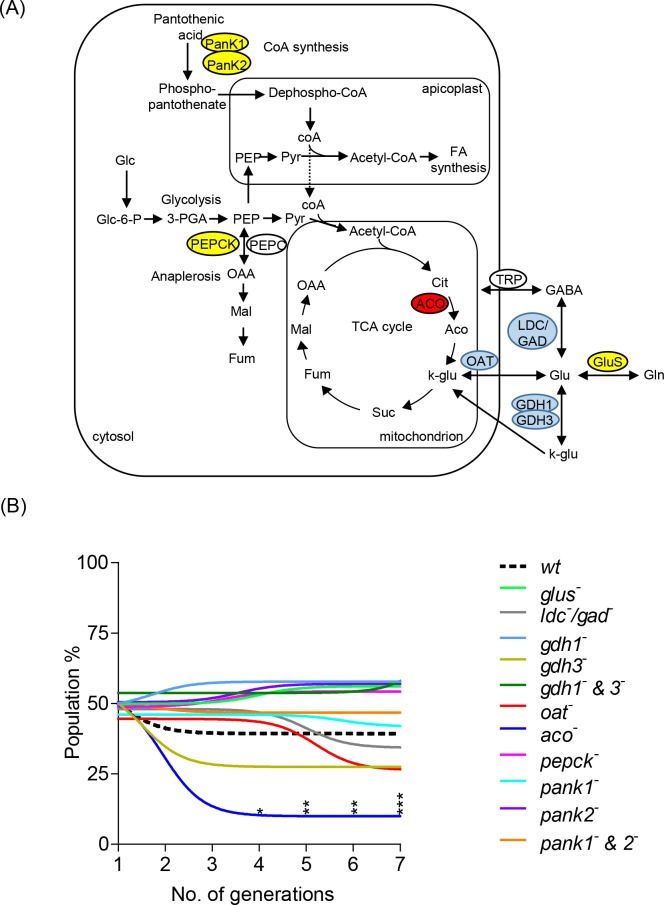
Genetic dissection of central carbon metabolism in asexual blood stages. (A) Schematic representation of central carbon metabolism in *P*. *berghei* showing genes encoding metabolic enzymes disrupted or discussed in this study. Abbreviations as for Fig 1 and: PanK1, pantothenate kinase 1; PanK2, pantothenate kinase 2; PEPC, phosphoenolpyruvate carboxylase; PEPCK, phosphoenolpyruvate carboxykinase; ACO, aconitase; OAT, ornithine amino transferase; TRP, putative GABA transporter; GDH1, glutamate dehydrogenase 1; GDH3, glutamate dehydrogenase 3; LDC, lysine decarboxylase/ GAD, glutamate decarboxylase; GluS, glutamate synthase. Disrupted genes coloured in blue have no effect on asexual or mosquito stage development, those coloured in red have no effect on asexual stage development but fail to make ookinetes and genes coloured in yellow have no effect on asexual stage development but show a strong phenotype in ookinete to oocyst transition with a block in transmission. See Fig 8 and S3 Table for summary. (B) *In vivo* growth assay of *P*. *berghei* mutants in mixed competition infections with wild type (wt) parasites over 7 generations. Coloured lines represent the non-linear fit of percentage of mutant parasites in the total parasite population (mixed with wt 50–50). Data representative of n = 3 independent biological replicates. P-value ***p < 0.0001, **p < 0.001, *p < 0.01 Two way ANOVA- Dunnett's multiple comparison test with wt control compared to mutant *P*. *berghei* parasites per generation. Also see S3 and S7 Figs.

In order to examine which, if any, of these metabolic pathways are essential for intracellular growth of different parasite stages, reverse genetic approaches were used to generate mutant lines lacking key enzymatic functions in GABA/glutamine metabolism, TCA metabolism, anaplerotic anabolism and CoA synthesis individually or in specific combinations (Fig 2A, S2 Fig). *In vivo* asexual growth of these mutant parasites was monitored using a single host competitive growth assay in the presence of wild type parasites (Fig 2B) as previously described [26]. *P*. *berghei* mutants lacking an NAD(P)H-dependent glutamate synthase (GluS), the enzyme responsible for the interconversion of glutamine to glutamate (*glus*^*-*^, PBANKA_1009500) had similar growth rates to wild type parasites, indicating that this enzyme is not essential and/or can be bypassed by salvage of glutamate from the RBC. Similarly *P*. *berghei* mutants lacking two of the three isoforms of glutamate dehydrogenase (GDH) (*gdh1*^*-*^ PBANKA_102620, *gdh3*^*-*^ PBANKA_122820 and the *gdh1*^*-*^ & *gdh3*^*-*^ double mutant) grew similarly to wild type parasites. Although the *gdh3*^*-*^ exhibited a slightly reduced growth rate, this was not significant. On the other hand, repeated attempts to generate clonal populations for *gdh2*^*-*^ (PBANKA_101400) were unsuccessful, indicating that this isoform is essential, presumably either because of its role in channelling glutamate into the TCA cycle and/or its role in regulating NADP/NADPH reducing equivalents. Viable *P*. *berghei* mutants lacking the putative lysine decarboxylase/glutamate decarboxylase (*ldc*^*-*^ */gad*^*-*^, PBANKA_100340) postulated to synthesise GABA from glutamate [28] were generated and grew at the same rate as wild type asexual parasites, indicating that the limited GABA shunt is not essential for asexual stages. Consistent with this conclusion, viable mutant parasite lines lacking the ornithine amino transferase (OAT), a putative GABA/glutamate transaminase which can recycle glutamate, were also obtained (*oat*^-^, PBANKA_010740) and these parasites grew only slightly less quickly than wild type. However, attempts to generate clonal populations of a mutant (*trp*^-^, PBANKA_030670) lacking a putative GABA transporter (TRP) [10] were unsuccessful, suggesting that this transporter may have a broader role than GABA uptake/efflux. Deletion of another amino transferase (PBANKA_030230) in *P*. *berghei* was previously shown to be refractory [26].

A *P*. *berghei* mutant (*aco*^*-*^, PBANKA_135520) lacking the TCA cycle enzyme aconitase (ACO), which is responsible for the isomeric conversion of citrate to isocitrate, was generated but exhibited a severe growth defect during asexual development (Fig 2B). *aco*^*-*^ parasites grew much slower than wild type parasites but were not completely outgrown and close scrutiny of these mutants showed that they have a prolonged asexual cycle (up to 4 h longer in *aco*^-^ than wild type, S3 Fig). On the other hand, disruption of the gene encoding phosphoenolpyruvate carboxykinase (PEPCK) which is antagonistic to PEPC, predicted to have a role in TCA cycle anaplerosis (*pepck*^*-*^, PBANKA_135590) had little effect on asexual parasite development. Interestingly, disruption of the two *P*. *berghei* pantothenate kinases (PANK1 and PANK2), required for phosphorylation of pantothenic acid and the synthesis of CoA, individually (*pank1*^*-*^, PBANKA_1022600 and *pank2*^-^, PBANKA_061140) or together (*pank1*^*-*^ *& 2*^*-*^), also had little impact on asexual parasite development (Fig 2B). Collectively, these findings suggest that, with the exception of ACO, several key enzymes associated with mitochondrial metabolism are not essential in *P*. *berghei* asexual blood stages and support the view that energy for intracellular survival and growth is primarily derived from glycolysis.

### Gametocytes and gametes rely mainly on glycolysis but also display dependence on glutamine metabolism

U-^13^C-glucose and U-^13^C^15^N-glutamine labelling studies were performed on *P*. *berghei* gametocytes generated from the gametocyte producer parent line, RMgm-164 [27]. Mature gametocytes were obtained from *P*. *berghei* infected mice, magnetically purified and incubated for 2 h at 37°C in minimal media containing U-^13^C-glucose or U-^13^C^15^N-glutamine prior to activation. To form gametes, gametocytes were then switched to minimal media containing the gametocyte activation factor, xanthurenic acid [29] and labelled carbon sources at 21°C (mimicking mosquito midgut conditions). Cells were harvested at 0, 10, 20 and 30 min time points and metabolism was rapidly quenched prior to metabolite extraction and quantification of ^13^C enrichment by GC-MS.

#### U-^13^C-glucose and U-^13^C^15^N-glutamine labelling during gametocyte activation

**Fig 3:**
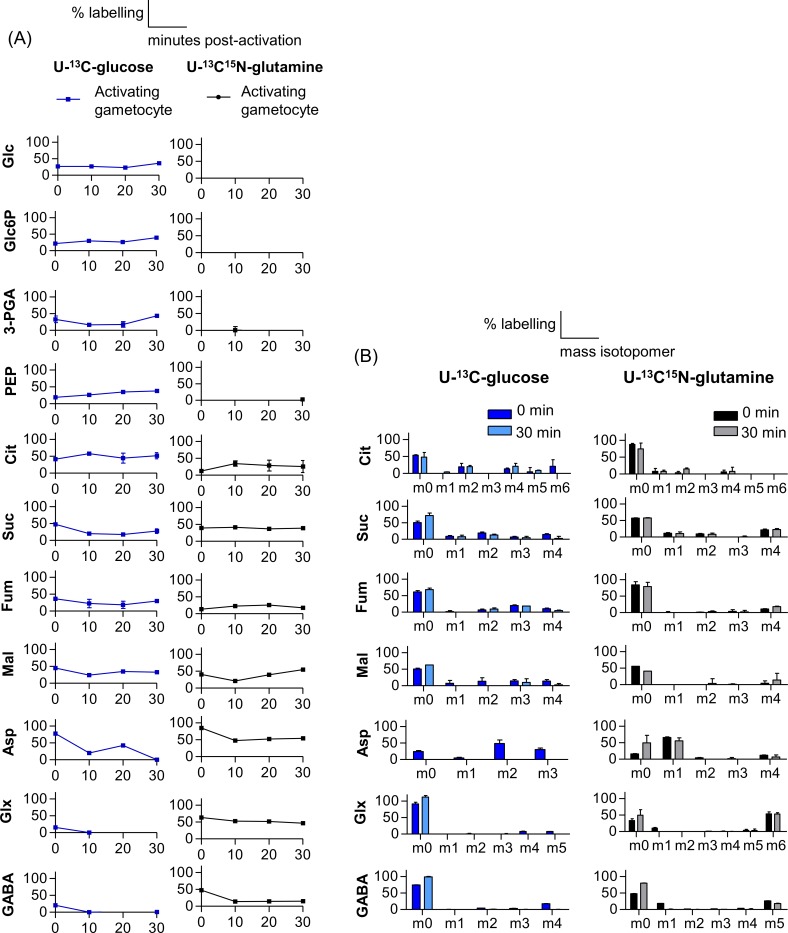
U-^13^C-glucose and U-^13^C^15^N-glutamine labelling of glycolytic and TCA cycle intermediates in *P*. *berghei* gametocytes during activation. (A) Gametocytes were activated and then metabolically labelled with U-^13^C-glucose and U-^13^C^15^N-glutamine for indicated times during activation (0, 10, 20, 30 min post activation). Percentage labelling (mol% containing one or more ^13^C carbons after correction for natural abundance) in indicated metabolites was determined by GC-MS. (B) Fractional labelling of TCA-cycle intermediates in unactivated gametocytes (0 min) and activated gametes (30 min post activation) cultured in the presence of U-^13^C-glucose and U-^13^C^15^N-glutamine. The x-axis indicates the number of ^13^C atoms in each metabolite (the ion used to analyse aspartate contains 3 of the 4 carbons as the 4-carbon fragment was below the limit of quantification). Due to the presence of a labelled nitrogen atom when labelling with U-^13^C^15^N-glutamine, the isotopologue analyses of nitrogen-containing metabolites include an isotope 1 dalton higher than the U-^13^C-glucose equivalent; i.e. aspartate (Asp, +4), glutamine/glutamate (Glx, +6) and ɣ-aminobutyric acid (GABA, +5). Error bars indicate SD of n = 3 biological replicates. Abbreviations are same as in Fig 1.

Metabolic labelling of both glycolytic and TCA cycle intermediates by U-^13^C-glucose was observed during gametocyte activation indicating the importance of glycolysis and entry of glycolytic pyruvate into the TCA cycle (Fig 3A). Isotopologue analysis of U-^13^C-glucose labelled gametocytes in unactivated gametocytes (0 min) and gametes (30 min) revealed +2 and +4 isotopologues of all TCA intermediates, indicating an active canonical TCA cycle (Fig 3B). +3 Isotopologues were also observed (especially in fumarate and aspartate), indicating the presence of inter-conversions with the PEPC. Labelled aspartate was present at 20 min post activation but disappeared after 30 min (Fig 3A) and hence was likely fully converted to malate and/or fumarate after this time by PEPC dependent intermediary carbon metabolism (ICM), since both of these metabolites have the +3 isotopologue as the main labelled ions at 30 min post-activation. Another possibility could be the utilisation of aspartate in pyrimidine biosynthesis to contribute to nucleotide pools which may be required in an activating gametocyte that is undergoing rapid endomitosis. Pyrimidine biosynthesis mutants have previously been shown to be defective at this stage [26].

As expected, label derived from U-^13^C^15^N-glutamine was incorporated into all TCA intermediates (Fig 3A) in activating/activated gametocytes consistent with the action of a canonical TCA cycle with glutamine as a carbon source. More than half the GABA was labelled in unactivated gametocytes fed U-^13^C^15^N-glutamine and this labelling decreased during the process of activation, suggesting a role of GABA/glutamine metabolism during this process. Isotopologue analysis of U-^13^C^15^N-glutamine labelled TCA cycle intermediates (Fig 3B) showed +2 and +4 isotopologues and the +4 labelling was much higher than that seen when parasites were labelled with U-^13^C-glucose, indicating that glutamine is the primary carbon source driving the TCA cycle.

#### *P*. *berghei* parasites deficient in key metabolic pathways form gametocytes but show reduced male gametogenesis

**Fig 4:**
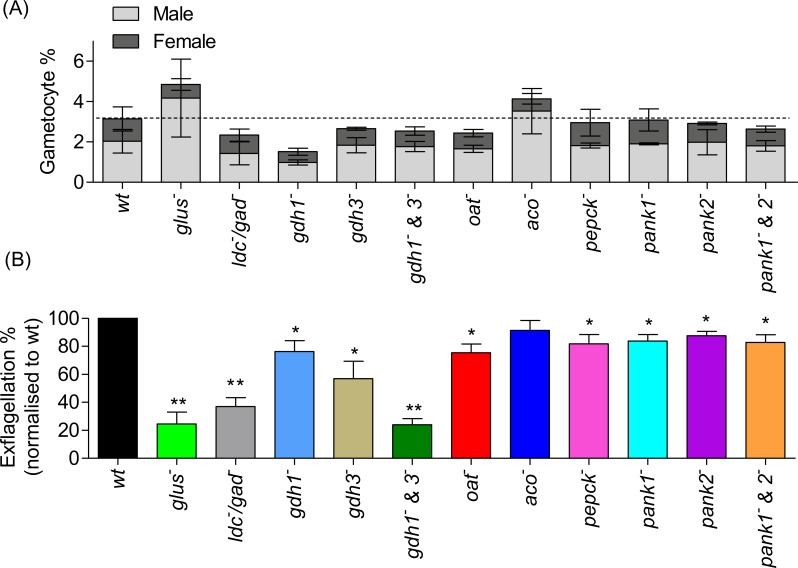
Gametocyte conversions and exflagellation in *P*. *berghei* mutant parasites. (A) Gametocyte conversion was observed during blood stage development in mutant *P*. *berghei* parasites over 5 days post infection by either using a wt parent line which expresses GFP in male gametocytes and RFP in female gametocytes (RMgm-164) with *P*. *berghei* mutants generated in the same genetic background and analysed using FACS determining the number of gametocytes in infected blood or by observing mature gametocytes in Giemsa stained smears. No significant difference was seen between wt and mutants parasites. Error bars indicate SD of n = 2 biological replicates. (B) Exflagellation (male gamete formation) in mutant *P*. *berghei* parasites normalised to wt in an *in vitro* activation assay. Error bars indicate SD of n = 3 biological replicates. P-values **p < 0.005, *p < 0.05 unpaired two tailed t-test compared to wt.

*P*. *berghei* mutant parasites (Fig 2A) were analysed for their ability to form (the correct ratio and number of) gametocytes in peripheral circulation and to see if disruption of any of these metabolic pathways had an effect on exflagellation (a measure of male gamete formation). None of the mutant parasites showed any significant difference compared to wild type parasites in the number of gametocytes produced or their male to female ratio (Fig 4A). However, a decrease in exflagellation compared to wild type was observed in a number of mutants (Fig 4B) including the *glus*^*-*^ (75% less than wild type, p < 0.005), *ldc*^*-*^*/gad*^*-*^ (63% less than wild type, p < 0.005) and *gdh1*^*-*^*&3*^*-*^ (76% less than wild type, p < 0.005) mutants. A very slight decrease was seen in all other mutants (p < 0.05) except the *aco*^*-*^ mutant which had wild type levels of exflagellation (Fig 4B). This is in contrast to *P*. *falciparum* where chemical [10] or genetic [11] disruption of aconitase leads to arrest in gametocytogenesis and male gamete formation (microgametogenesis) [11]. The data suggest that glutamine/GABA metabolism plays an important, if not critical role in exflagellation during microgametogenesis in *P*. *berghei*.

#### Glycolysis is essential for male gametogenesis

**Fig 5:**
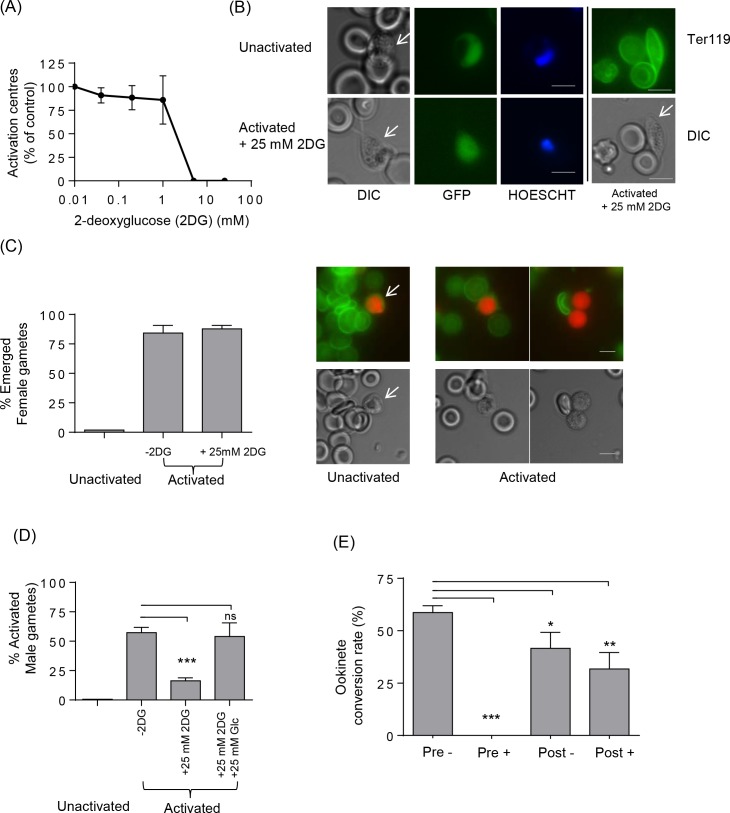
2-Deoxyglucose (2DG) specifically inhibits male gametocyte activation. (A) Plot showing the number of activation centres observed in cultures containing 2DG compared to control in ookinete media alone. Error bars indicate SD of n = 6 biological replicates. (B) Microscopy images of male gametocytes after activation in the presence of 2DG. GFP expressing male gametocytes of the RMgm-164 line exhibited a characteristic shape (arrowed in left panel) and were positive for the red blood cell marker Ter119 (arrowed in right panel) indicating that they had not emerged from the host red blood cell. Scale bar = 5 μm. (C) Left Panel: Female gametocytes emerged in the presence of 2DG as counted under fluorescent microscope after counterstaining with a fluorescent conjugated red blood cell marker Ter119. Error bars indicate SD of n = 6 biological replicates. Right panel fluorescent microscope images showing the red fluorescent female parasites of the RMgm-164 line with the green FITC-Ter119 red blood cell membrane marker (top panel), DIC image (lower panel) for unactivated (arrowed, left) and activated emerged females (right). (D) Plot showing the percentage of male gametes activated in cultures containing 25 mM 2DG (+25 mM 2DG) is reduced significantly compared to the control (-2DG) and this can be rescued by addition of an equimolar amount of glucose (+25 mM Glc). Error bars indicate SD of n = 4 biological replicates. P-value ***p < 0.0005, **p < 0.005, *p < 0.05, paired two tailed t-test compared to -2DG. (E) Conversion to ookinetes after 2DG treatment is inhibited. Parasites were activated then 2DG (25 mM) was added 30 min post activation (post). Alternatively parasites were incubated in BSA enriched PBS containing 25 mM 2DG for 30 min before activation in media also containing 2DG (pre). Conversion rates were calculated after 21 h post activation. Error bars indicate SD of n = 2 biological replicates. P-value ***p < 0.0005, **p < 0.005, *p < 0.05, paired two tailed t-test compared to Pre-.

Our labelling analyses and those of others [10,11] indicated that glycolysis and glutamine catabolism both feed the TCA cycle in *Plasmodium* gametocytes and that glutamine catabolism had a significant role in microgametogenesis in *P*. *berghei*. Glycolysis is required for microgamete motility [30] and the *Plasmodium* hexose transporter is essential for both asexual blood stage growth and microgametogenesis [31,32]. Disruption of mitochondrial α-ketoglutarate dehydrogenase in *P*. *falciparum*, however does not affect microgametogenesis [11]. To further examine the extent to which gametes are dependent on glycolysis or whether they can switch to mitochondrial respiration during microgametogenesis, these stages were cultured in the presence of 2-deoxyglucose (2DG), a substrate for hexokinase which cannot be further catabolized in the glycolytic pathway [33]. In a haemocytometer based counting assay, microgametogenesis was found to be inhibited by 2DG in a dose-dependent manner and no microgametes emerged from the red blood cells at concentrations of 2-deoxyglucose above 8 mM (Fig 5A and 5B). Microgametogenesis was not rescued by glutamine in the culture media, consistent with our observations of the important but not essential role of glutamine catabolism in this process. In other eukaryotes, 2DG may have secondary effects on other pathways [34,35] which may also be essential for parasite survival. However, to test whether glycolysis inhibition was the main driver of reduction in microgametogenesis, we added equimolar amounts (25 mM) of glucose in the presence of 2DG and using a flow cytometry based high throughput assay, found that it was possible to rescue the inhibition of microgametogenesis by 2DG (Fig 5D). However, the inhibitory effect of 2DG on *P*. *berghei* gametogenesis was sex-specific since female (macro) gametes emerged from the red blood cells in the presence of 2DG (Fig 5C). Moreover, as seen when the hexose transporter was inhibited [31], pre-incubation of gametocytes with 2DG had a greater inhibitory effect on conversion to ookinetes than if the reagent was added after activation (Fig 5E). This sex-specific effect of 2DG can be exploited to produce populations of fertile activated female gametes for either further analysis or genetic crossing experiments and demonstrates that male gametogenesis (but not female gametogenesis) is reliant on glycolysis.

### Developing ookinetes have an active TCA cycle fed by both glucose and glutamine

Gametocytes from the gametocyte producer parent line, RMgm-164 [27] as described above and RMgm-15 (Pb137, *p48*^*-*^*/45*^*-*^), which produces viable female gametes but non-viable male gametes [36], were activated and cultured for 21 h at 21°C. RMgm-164 produces fertilised female gametes which mature into ookinetes; therefore RMgm-15 was used to discriminate between activated, unfertilised and fertilised female gametes at the metabolic level. Gametes were labelled with U-^13^C-glucose or U-^13^C^15^N-glutamine; samples were collected after 10 h (retort stage) and 21 h (mature ookinetes) and magnetically purified at 21°C before rapidly quenching metabolism, metabolite extraction and quantification of ^13^C enrichment by GC-MS.

#### U-^13^C-glucose and U-^13^C^15^N-glutamine labelling during ookinete development

**Fig 6:**
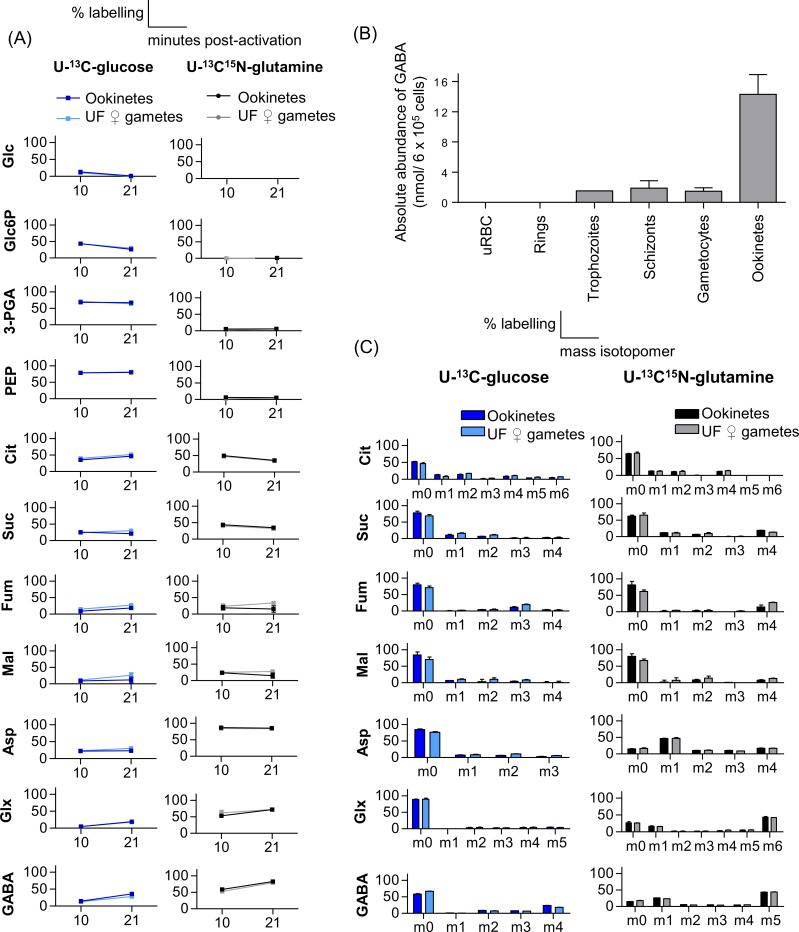
U-^13^C-glucose and U-^13^C^15^N-glutamine labelling of glycolytic and TCA cycle intermediates in *P*. *berghei* ookinetes and unfertilised (UF) female gametes. (A) *P*. *berghei* ookinetes and unfertilized gametes were metabolically labelled with U-^13^C-glucose and U-^13^C^15^N-glutamine for 10 hr and 21 hr post activation, and percentage labelling (mol. % containing one or more ^13^C carbons after correction for natural abundance) was determined by GC-MS. (B) Absolute abundance of GABA in uninfected erythrocytes (uRBC) and *P*. *berghei* infected erythrocytes (iRBC) at ring, trophozoite, schizont, gametocyte and ookinete stages in nmol per 6 × 10^5^ cells (normalised to magnetically purified parasite numbers). (C) Fraction labelling of TCA-cycle isotopologues in mature ookinetes and unfertilised (UF) female gametes at 21 h post activation cultured in the presence of U-^13^C-glucose and U-^13^C^15^N-glutamine. The x-axis indicates the number of ^13^C atoms in each metabolite (the ion used to analyse aspartate contains 3 of the 4 carbons as the 4-carbon fragment was below the limit of quantification in our GC-MS data). Due to the presence of a labelled nitrogen atom when labelling with U-^13^C^15^N-glutamine, the isotopologue analyses of nitrogen-containing metabolites include an isotope 1 dalton higher than the U-^13^C-glucose equivalent; i.e. aspartate (Asp, +4), glutamine/glutamate (Glx, +6) and ɣ-aminobutyric acid (GABA, +5). Error bars indicate SD of n = 3 biological replicates. Abbreviations are same as in Fig 1.

U-^13^C-glucose labelling demonstrated that glycolysis is highly active and feeds the TCA cycle in both (developing) ookinetes and activated, unfertilised female gametes. All glycolytic and TCA cycle intermediates were found to be strongly labelled (Fig 6A). Isotopologue analysis of metabolites from U-^13^C-glucose labelled parasite stages revealed +3 labelled fumarate and malate indicating active ICM (Fig 6C). U-^13^C^15^N-glutamine labelling confirmed that the active TCA cycle could be sustained by glutamine catabolism in ookinetes and unfertilised female gametes (Fig 6A U-^13^C^15^N-glutamine fed ookinetes contained expected +2 and +4 isotopologues supporting the operation of a canonical TCA cycle during ookinete development. The higher proportion of +4 ions again indicated that carbon flux through glutamine dominates over flux from glucose. Intriguingly, both labelling (Fig 6A) and the absolute levels of GABA (Fig 6B) in mature ookinetes (14.3 ± 2.6 nmol/ 6 × 10^5^ cell equivalents) were found to be elevated compared to other life cycle stages examined (~ 8-fold higher than in asexual stages and gametocytes). GABA isotopologues in both mature ookinetes and unfertilised, activated female gametes (Fig 6C), indicated significant flux of glutamate into GABA synthesis.

#### *P*. *berghei* parasites deficient in key metabolic pathways show reduced metabolic flexibility during mosquito stage development

**Fig 7:**
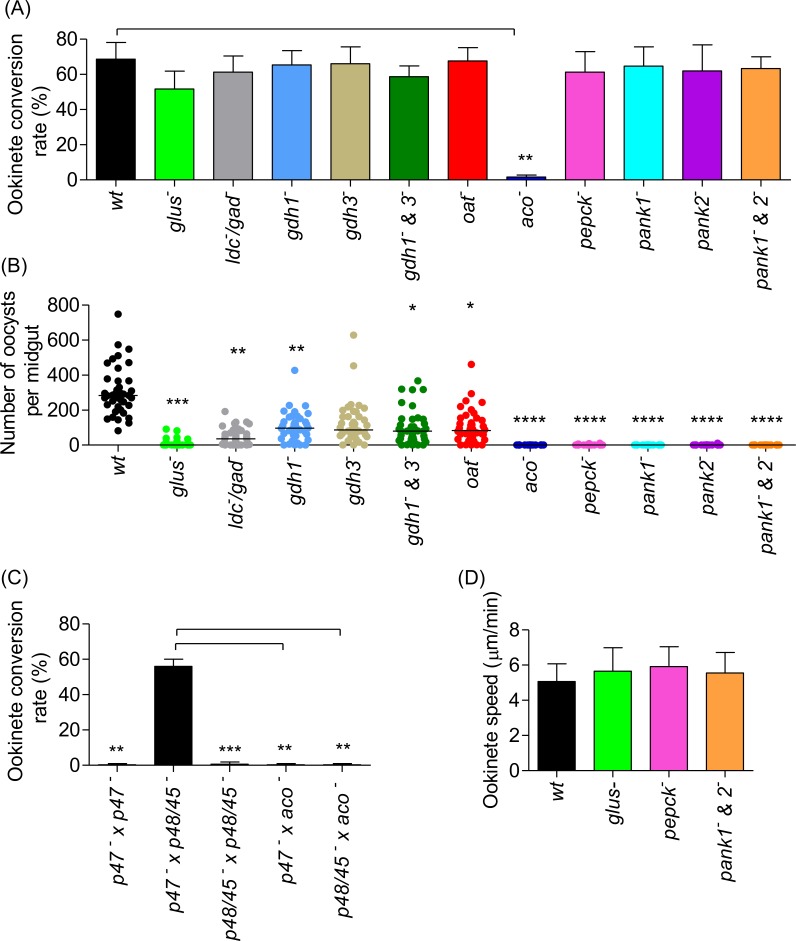
Mosquito stage development of *P*. *berghei* mutant parasites. (A) *In vitro* ookinete conversion of mutant *P*. *berghei* parasites as compared to wt. The error is given as the SD of n = 3 independent biological replicates. P-value **p < 0.005 unpaired two tailed t-test compared to wt. (B) Number of mature oocysts at 7–12 days post-*P*. *berghei* mutant parasite-infected blood feed in mosquito mid guts. n = 40 mosquitoes cumulative of two independent biological replicates. P-values ****p < 0.00005, ***p < 0.0005, **p < 0.005, *p < 0.05 unpaired two tailed t-test compared to wt. (C) *in vitro* ookinete conversion assay to measure fertility of *aco*^-^ *P*. *berghei* gametocytes. Fertility of *aco*^-^ *P*. *berghei* gametocytes was analysed by their capacity to form ookinetes by crossing gametes with RMgm-348 (Pb270, *p47*^*-*^) which produces viable male gametes but non-viable female gametes and RMgm-15 (Pb137, *p48/45*^*-*^) which produces viable female gametes but non-viable male gametes. *p47*^*-*^ and *p48/45*^*-*^ self crosses serve as negative controls and *p47*^*-*^ x *p48/45*^*-*^ cross is the positive control. *aco*^-^ cross with either *p47*^*-*^ or *p48/45*^*-*^ did not produce any ookinetes. The error is given as the S.D. of n = 2 independent biological replicates. P values ***p<0.0005, **p<0.005 unpaired two tailed t-test compared to cross *p47—*x *p48/45 –*. (D) Ookinete motility assay. Mature ookinetes were embedded in matrigel and tracks were constructed on Image J. Displacement in 10.5 min was calculated for ookinetes moving in a straight line and represented as speed of motility in μm/min. (n = mean 40 ookinetes).

The ability of *P*. *berghei* metabolic mutants to develop in the mosquito and complete transmission was assessed by their ookinete conversion rate, ookinete to oocyst transition, and formation of infectious salivary gland sporozoites. None of the GABA/glutamine metabolism mutants showed any defect in ookinete conversion (Fig 7A). By contrast 30-fold fewer oocysts were produced in *glus*^*-*^ mutants (p < 0.0005) (Fig 7B, S4 Fig). These *glus*^*-*^ mutants did not produce any salivary gland sporozoites (S5 Fig) and failed to transmit through the mosquito. The *ldc*^-^/*gad*^-^ (p < 0.0005), *gdh1*^-^(p < 0.005), *gdh1*^*-*^*&3*^*-*^ (p < 0.05) and *oat*^-^ (p < 0.05) mutant lines also displayed a significant reduction in oocyst numbers (Fig 7B, S4 Fig) but were still capable of producing sporozoites in mosquito salivary glands (S5 Fig) and completing transmission through the mosquito. Mosquito transmission of these mutant lines to naïve mice resulted in the generation of blood stage asexual forms in 48–72 h with the same kinetics as the wild type parental line.

Despite possessing similar exflagellation rates to wild type parasite (Fig 4B), ookinete conversion was found to be severely affected in *aco*^-^ parasites (Fig 7A). To determine if this defect was sex-specific, genetic crosses of *aco*^*-*^ parasites were performed with *P*. *berghei* lines RMgm-348 (Pb270, *p47*^-^, which produces viable male gametes but non-viable female gametes) and RMgm-15 (Pb137, *p48/45*^*-*^, which produces viable female gametes but non-viable male gametes)[36]. Surprisingly, given the maternal inheritance of the mitochondrion, *aco*^*-*^ parasites were found to produce severely reduced numbers of ookinetes in both crosses (Fig 7C), suggesting that all gametes are affected in the absence of a complete TCA cycle. Not surprisingly, oocysts (Fig 7B, S4 Fig) and salivary gland sporozoites (S5 Fig) were not observed in *aco*^-^ parasites and transmission was found to be completely blocked in these mutants. The *pepck*^-^, *pank1*^*-*^, *pank2*^*-*^, and *pank1*^*-*^*&2*^*-*^ mutant parasites produced wild type levels of ookinetes (Fig 7A). They were, however, severely compromised in producing mature oocysts (Fig 7B, S4 Fig) and salivary gland sporozoites (S5 Fig), leading to a complete block in transmission. The motility of mutant ookinetes which failed to complete transition to oocysts was not affected (Fig 7D), implying that the phenotype observed in ookinete to oocyst transition was further downstream of the motile ookinete stage for these mutants.

## Discussion

**Fig 8:**
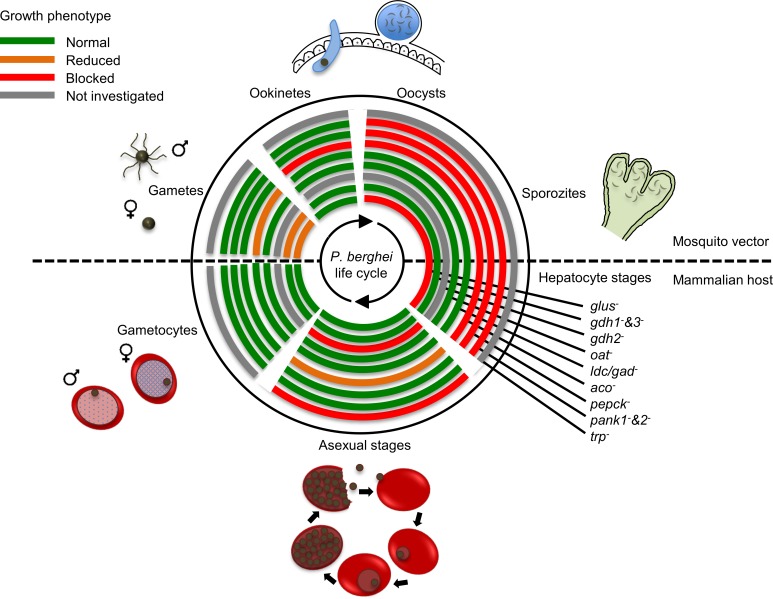
A schematic representation of the life cycle of *P*. *berghei* and the effect of disrupting metabolic genes in this study.

The malaria parasite invades and replicates within a number of different host cell types during its development in the mammalian host and mosquito vector. While it is presumed that the different *Plasmodium* developmental stages have different metabolic requirements as well as encounter different nutrient conditions in each of these niches, comparatively little is known about the stage-specific changes in parasite metabolism. In this study we show that differentiation of *P*. *berghei* vertebrate (asexual RBC stages and gametocytes) and mosquito (gametes and ookinetes) stages is linked to marked changes in central carbon metabolism and dependency on key enzymes in TCA cycle and amino acid metabolism (Fig 8 and S3 Table). These changes in carbon metabolism appear to be hard-wired into the differentiation program and are likely required to support the particular energy and growth requirements of each stage.

### Stage-specific changes in *P*. *berghei* central carbon metabolism

Comprehensive ^13^C-labeling experiments confirmed that all *P*. *berghei* stages tested, including asexual RBC stages, gametocytes, gametes and ookinetes, exhibit high rates of glucose utilization which is required to drive anaerobic glycolysis (for ATP synthesis) and the pentose phosphate pathway, as well as pathways of sugar nucleotide and glycoconjugate biosynthesis. This dependence on glucose uptake and catabolism, which was confirmed in the case of microgametogenesis using the inhibitor 2-deoxyglucose, is not surprising as *Plasmodium* spp. lack the key gluconeogenic enzyme fructose 1,6-bisphosphatase and are therefore completely dependent on glucose uptake for *de novo* synthesis of hexose phosphates. Against this background of constitutive glucose utilization, we observed a progressive increase in the extent to which pyruvate was further catabolized in the TCA cycle following the differentiation of *P*. *berghei* asexual RBC stages to gametocytes and subsequently to mosquito stages (gametes and ookinetes) (Figs 1, 3 and 6). Consistent with recent studies in *P*. *falciparum* [10,11,17] and *P*. *berghei* [13], we showed that pyruvate flux into the TCA is very low in *P*. *berghei* asexual RBC stages and that the low but detectable flux through the TCA cycle in these stages is largely maintained by the uptake and catabolism of glutamine (glutaminolysis) and a partial GABA shunt. The low overall flux of pyruvate and glutamine into the TCA cycle of *P*. *berghei* asexual stages suggests that this plays a minor role in ATP synthesis and may be required to maintain redox balance and membrane potential of the mitochondria.

A proportion of asexual RBC stages differentiate to gametocytes, which are required for transmission and production of gametes in the mosquito midgut. Mature circulating gametocytes are cell cycle-arrested and sexually dimorphic, eventually producing male and female forms that have different biological activities upon transfer to the mosquito midgut environment. Labelling studies indicated that mature *P*. *berghei* gametocytes exhibit elevated levels of TCA cycle activity sustained by increased catabolism of pyruvate and glutaminolysis (Fig 3), as has also been observed in *P*. *falciparum* gametocytes [10]. The elevated TCA cycle flux in the gametocyte stages of both murine and human malaria parasites is associated with enlargement of the mitochondrion and increased cristae formation in both male and female gametocytes [37,38].

Following transmission to the mosquito vector, male and female gametocytes initiate quite distinct developmental programs. Most strikingly, female gametocytes retain a mitochondrion, while this organelle is largely degenerate in the mature male gametocyte [20,39–41]. Upon activation, the male gametocyte initiates three rounds of energy intensive rapid nuclear replication and packages the resulting nuclei into eight male gametes [42,43]. Our labelling experiments demonstrated that these motile parasite stages exhibit high rates of glycolysis which are likely to be the major source of ATP for male microgamete motility [30,31] and exflagellation. Inhibition of glycolysis with 2DG prevented exflagellation and microgametogenesis, but did not affect female gamete maturation. While female gametes may be able to switch to using glutamine as an alternative (short term) carbon source for ATP synthesis under these conditions, the absence of a functional mitochondrion and TCA cycle in male gametes prevent such a switch. The absence of an active TCA cycle metabolism in male gametes may also make these stages more dependent on enzymes involved in the interconversion of exogenously scavenged glutamine and glutamate (which is normally synthesized from TCA cycle intermediates in other stages) consistent with reduced male gamete production in the *P*. *berghei* mutants lacking genes involved in GABA and glutamine catabolism (described below) (Fig 4B).

In the mosquito mid-gut, male and female gametes fuse to form an apolar zygote which further develops into a motile polar ookinete. Ookinetes must be able to sustain high motility in order to transit across the gut epithelial layer and escape from the hostile environment of the blood meal and the mosquito digestive tract. Our data demonstrate that maturing ookinetes are metabolically active and can co-catabolize glucose and glutamine via the TCA cycle. Interestingly, glutamine was the predominant carbon source utilized in the TCA cycle indicating a preference for glutaminolysis for ATP synthesis. This may indicate metabolic compartmentalization of glucose utilization and preferential direction of hexose phosphates into essential pathways such as the pentose phosphate pathway and nucleotide synthesis. Unfertilized activated female gametes also exhibited a similar metabolic programme to fertilized ookinetes, suggesting that fertilization is not a prerequisite for these metabolic changes.

### Role of host cell central metabolism in supporting the growth of intracellular *P*. *berghei* parasites

Even though glycolysis was confirmed to be present in uRBC, U-^13^C-glucose was found be consumed in iRBC much more rapidly than in uRBC (Fig 1A). This is in line with previous studies [44] and pointed towards the rapid turnover of glycolytic end products in iRBC to downstream metabolites via the TCA cycle and/or intermediary carbon metabolism or for rapid biomass generation to help in proliferative schizogony [45]. Although pre-existing pools of glucose in the host cell can contribute to glycolysis in the parasite, the direct role of the host cell glycolytic pathway in development of intracellular parasites is unclear as the parasite glycolytic machinery is well established [46] and is supported by increased internalisation of glucose into the infected cell owing to expression of glucose transporters on the surface of the infected cell [32]. However, the ability of the host erythrocyte to maintain its own ATP pools via glycolysis ensures its viability and supports the survival of the resident parasite. Moreover, the parasite can also supply the host cell with ATP in order to improve this process [47].

It is notable that host reticulocytes also retain residual mitochondrial metabolism and TCA cycle flux is fed by glutaminolysis, although this cycle appears to function primarily in reductive mode, with conversion of α-ketoglutarate (derived from glutamine) to citrate. Reductive carboxylation has been observed in cells with functionally compromised mitochondria [48] such as occurs in maturing reticulocytes [49]. In other systems, reductive metabolism facilitated by glutaminolysis has been shown to generate lipogenic acetyl-CoA [50,51]. The function of a reductive TCA cycle in *P*. *berghei*-infected reticulocytes and its impact on the intracellular parasite is unknown at this stage and it would be interesting to investigate whether host cell generated two-carbon units from this pathway contribute to parasite metabolism.

### Functional requirement for TCA cycle and glutamine metabolism in different life cycle stages

In order to determine which, if any, of these stage-specific changes in *P*. *berghei* central carbon metabolism were important for parasite development and mosquito transmission, mutant lines lacking aconitase (ACO), a key enzyme required for catabolism of pyruvate in the TCA cycle and several enzymes involved in glutamate/GABA metabolism (GluS, GDH 1 and 3, LDC/GAD, OAT) were generated (S3 Table). Interestingly, a delayed growth phenotype was seen in *aco*^*-*^ mutants in the asexual RBC stages which have the least active mitochondrial TCA cycle of all the stages and little effect was seen on the development of *P*. *berghei* gametocytes and gametes. Any further development of these mutants was blocked in the mosquito. These findings suggest that any loss of glycolytic pyruvate catabolism in the *P*. *berghei* TCA cycle is compensated for by either a much larger steady-state of pre-existing TCA cycle metabolites or increased glutaminolysis. The slow growth phenotype seen in *P*. *berghei aco*^*-*^ mutant parasites could be caused by an imbalanced redox state in the mitochondria due to decrease in NADPH production. Furthermore, species-specific differences may exist to the extent that glutaminolysis compensates for loss of glucose catabolism in the TCA cycle. Specifically, disruption of the gene encoding aconitase [11] or chemical inhibition of aconitase [10] in *P*. *falciparum* inhibited gametocyte maturation. While this apparent requirement for aconitase for *P*. *falciparum* but not *P*. *berghei* gametocytogenesis may reflect species-specific differences in the metabolic networks of these parasites, it may also result from the different host cell environments occupied by the gametocytes of each species. Indeed we have previously shown that *P*. *berghei* is more permissive to other metabolic gene knock-outs than *P*. *falciparum* due to differences in their tropism for different host cells [26]. Moreover, it has been reported [14] that TCA cycle disruption by deletion of a gene of flavoprotein (Fp) subunit *Pbsdha* (PBANKA_051820), one of the four components of complex II, a catalytic subunit for succinate dehydrogenase activity also does not affect gametocyte maturation in *P*. *berghei* and the point of disruption (whether it is before the entry of alpha-ketoglutarate or after) does not seem to be crucial. As suggested in the *P*. *falciparum* aconitase disruption study [11], accumulation of citrate to toxic levels which seems to hinder the process of gametocytogenesis in *P*. *falciparum* over 7–10 days in *aco*^-^ mutants does not happen in a 48 hour asexual life cycle of *P*. *falciparum*. As *P*. *berghei* has even shorter blood stage developmental stages (24 hour asexual life cycle, 30 hours for gametocyte maturation), cytotoxic effects of accumulating citrate may well be avoided. Gametocytes thus formed seem to be able to activate and form gametes as glycolysis is unaffected in these mutants but failed to develop further in the mosquito. Sexual crosses revealed that this defect was not sex specific and both male and female gametes were affected. The increased reliance of extra-cellular stages on a more efficient process of TCA cycle and oxidative phosphorylation for energy generation in the mosquito in the absence of the glucose rich environment of the mammalian host, halts its growth due to disruption of TCA metabolism in the *aco*^-^ mutant as seen in other studies [11,13,14].

Disruption of glutamate synthase (GluS), the enzyme up-stream of glutamate dehydrogenases (GDHs) responsible for the reversible interconversion of glutamate and glutamine (with concomitant utilization/production of α-ketoglutarate and NAD/NADPH) also had no effect on the growth of asexual RBC stages, gametocyte development and ookinete motility, but resulted in a defect in exflagellation, reduced oocyst production and an effective block in sporozoite production and mosquito transmission. We were unable to generate *P*. *berghei* mutant clones lacking GDH2, an apicoplast-located enzyme responsible for conversion of glutamine to α-ketoglutarate. This isoform of GDH may contribute to mitochondrial metabolism through transfer of intermediates between closely linked apicoplast and mitochondrion [52,53]. Another essential role of GDH2 could be the production of NADPH for type II fatty acid synthesis, isoprenoid synthesis or ferredoxin reduction for maintaining the redox balance in the absence of a pentose phosphate pathway or a photosystem in this plant-derived organelle [54]. The deletion of the other two cytosolic GDHs (GDH1 and GDH3), individually or in combination, had no major impact on parasite development and is consistent with the findings in *P*. *falciparum* [55]. These results highlight the importance of glutamine catabolism, either for glutamate synthesis and/or catabolism in the TCA cycle in these latter stages. Consistent with this conclusion, previous studies have shown that defects in the TCA cycle enzymes downstream of the entry of glutamate carbon skeletons (i.e. succinate dehydrogenase in *P*. *berghei* [14] and α-ketoglutarate dehydrogenase in *P*. *falciparum* [11]) were also associated with impaired development of ookinetes and ookinete to oocyst transition. The absence of a distinct phenotype in other stages of *P*. *berghei glus*^-^ mutant may reflect the extent to which glutamine-dependent transamindases can bypass the need for glutamate synthase and/or the availability of high glucose levels in blood, which may not be accessible in the mosquito vector.

We have previously shown that glutamate can be converted to GABA via a glutamate decarboxylase (GAD) (annotated as a lysine decarboxylase (LDC) in *Plasmodium* [10]) in the related apicomplexan parasite, *Toxoplasma gondii* [28]. In *Plasmodium* spp., GABA may be further converted to succinate semialdehyde by mitochondrial transaminases, providing a mechanism for balancing the levels of pyruvate and alanine. However *Plasmodium* spp., unlike *T*. *gondii*, appear to lack the next enzyme in a canonical GABA shunt that converts succinate semialdehyde to succinate which would be required for catabolism of GABA in the TCA cycle) [28,46]. As such, it is unlikely that GABA catabolism acts as a shunt to by-pass key reactions in the TCA cycle down-stream of α-ketoglutarate. Transcriptomic studies have shown that *ldc/gad* mRNA is elevated in *P*. *berghei* gametocytes [56]. Disruption of *ldc*/*gad* in *P*. *berghei* did not affect asexual RBC stages and gametocyte formation, although exflagellation was reduced. Nevertheless, *ldc*^*-*^/*gad*^-^ parasites could be transmitted through the mosquito, suggesting that GABA dependent transamination reactions in the mitochondrion are dispensable for *P*. *berghei* transmission. Curiously, it was not possible to obtain a clonal population for a *P*. *berghei* mutant lacking the putative GABA transporter (PBANKA_030670) [10]. This suggests that this transporter may be involved in transporting other substrates or that loss of transport may lead to toxic build-up of GABA in all stages.

### Enzymes involved in intermediary carbon metabolism and CoA synthesis are also important for ookinete to oocyst transition

The enzymes phosphoenolpyruvate carboxylase (PEPC) and phosphoenolpyruvate carboxykinase (PEPCK) play important roles in maintaining the levels of cytoplasmic pools of pyruvate, PEP and C4 dicarboxylic acids [7,12,26]. Transcription of *pepck* is up-regulated in *P*. *falciparum* gametocytes and zygotes [57] and *P*. *berghei* gametocytes and ookinetes [56] suggesting a role in mosquito stage development of the parasite. We demonstrated that PEPCK is not essential in blood stages and that *pepck*^*-*^ mutants also exflagellated to form wild type-like gametes and gave comparable ookinete conversion rates. Furthermore, *pepck*^*-*^ ookinetes were motile, although they failed to develop into mature oocysts and subsequent sporozoites, thus blocking transmission. PEPCK-derived PEP can be converted to pyruvate in the cytosol by the glycolytic enzyme, pyruvate kinase 1 [58] and be further catabolized in the mitochondrion TCA cycle [13] generating the ATP required for multiple rounds of DNA replication in sporogony. Alternatively it can enter the apicoplast where it can be converted to pyruvate and acetyl-CoA by pyruvate kinase 2 [59] and apicoplast-resident pyruvate dehydrogenase [60], respectively (Fig 2A). Apicoplast-derived acetyl-CoA is required for *de novo* fatty acid synthesis during sporozoite development in the mosquito by the FASII pathway in *P*. *falciparum* [61] and an unidentified route in *P*. *berghei* [62–64].

Pantothenic acid is the precursor for CoA, and essential co-factor for the oxidation of pyruvate (both in the mitochondrion and the apicoplast) and the substrate for acetyl-CoA synthesis. In *P*. *falciparum* it has been shown that structural analogues of pantothenate are growth inhibitors of the asexual blood stages *in vitro* [65,66]. In *P*. *berghei*, deletion of both pantothenate kinase 1 and 2 (*pank1 & 2*) enzymes (either individually or in tandem) generated mutants that grew similarly to wild type in the asexual developmental stages, but showed a defect in the ookinete to oocyst transition. The synthesis of CoA is not identical across the *Plasmodium* genus: the avian malaria parasite, *P*. *lophurae*, is incapable of *de novo* CoA synthesis and scavenges CoA from the host erythrocyte whereas both *P*. *falciparum* and *P*. *berghei* can synthesise CoA *de novo* [67]. However, *P*. *berghei pank* mutants may also scavenge CoA from the host reticulocytes since these cells are known to support the growth of metabolically-compromised *P*. *berghei* parasites [26], whilst *P*. *falciparum*, with its dependence primarily on mature RBCs *in vitro*, would lack such exogenous supplies. The CoA synthesis pathway in the parasite terminates in the apicoplast as the last enzyme of this pathway, dephospho-CoA kinase, is localised to this organelle [68]. It is possible that the CoA required for oxidation of pyruvate to power the TCA cycle (during mosquito stage development) or the CoA required for fatty acid synthesis (in oocyst sporogony) is compromised in the *pank* mutants, thereby arresting the ookinete to oocyst transition. Expression of both *pank1* and *pank2* is maximal in the ookinete stage in *P*. *berghei* [56] supporting the notion that CoA biosynthesis is important during transmission of *P*. *berghei*. As a number of inhibitors of this pathway are currently being investigated for asexual inhibition of the malaria parasite [65,66,69–71], these studies should be extended to assess the transmission-blocking capacity of these inhibitors.

In summary, we have assessed the importance of central carbon metabolism in different blood and mosquito stages of the rodent malaria parasite *P*. *berghei* using ^13^C-stable isotope labelling, targeted metabolomics and reverse genetics. We show that these parasites rely on glucose metabolism throughout the life cycle, while co-utilized glutamine is the major driver of TCA metabolism and crucial to the development of the extra-erythrocytic ookinete stage and ookinete to oocyst transition. Both intermediary carbon metabolism and CoA synthesis are also important for ookinete to oocyst transition with a possible role in oocyst sporogony. This study adds to the existing knowledge of stage specific metabolism in the malaria parasite and also points at key differences between different *Plasmodium* species.

## Materials and Methods

### Ethics Statement

All animal work was approved by the University of Glasgow’s Animal Welfare and Ethical Review Body and by the UK’s Home Office (PPL 60/4443). The animal care and use protocol complied with the UK Animals (Scientific Procedures) Act 1986 as amended in 2012 and with European Directive 2010/63/EU on the Protection of Animals Used for Scientific Purposes.

### Infection of laboratory animals with *P*. *berghei* parasites

For mouse infections, female Theilers Original (TO) outbred mice of body weight 26–30 g were used. Cryopreserved blood stages were thawed at room temperature and 0.02–0.5 mL of the suspension was injected intraperitoneally into a mouse. For infection with blood stages obtained from an infected mouse (mechanical passage), one droplet of tail blood (5 μL) was collected from an infected animal with a parasitaemia of 5–15% in 10 mL PBS and 0.1 mL of the suspension was injected intraperitoneally into a mouse. On day 4–7 after injection the parasitaemia increased from 0.1 to 5–20%. On day 4 or 5 after injection the parasitaemia ranged between 0.5–3%.

### Asexual cultures of *P*. *berghei*

Unlabelled *P*. *berghei* cultures were maintained for one cycle using standard methods. Minimal medium (S1 Table) was used as the growing medium. A comparative analysis of growth of all *P*. *berghei* stages *in vitro* in our minimal media showed growth dynamics similar to RPMI1640 (S6 Fig). Flasks were gassed for 30 sec with a gas mix containing 5% CO_2_, 5% O_2_, and 90% N_2_ and incubated overnight at 37°C on a shaker at a minimal speed just to keep the cells in suspension.

### Generation of knockout parasites and cloning

*P*. *berghei* schizonts (from lines ANKA cl15cy1, RMgm-7 which expresses GFP constitutively under the *eef1a* promoter and from line RMgm-164 which expresses GFP in male gametocytes and RFP in female gametocytes) were transfected with linear DNA constructs containing the *yfcu-hdhfr* selectable marker flanked by homology arms (generated using primers in S2 Table) corresponding to 5’UTR and 3’UTR of the orf/catalytic domains of the gene of interest respectively for double crossover homologous recombination, injected intravenously in female TO mice and selected by pyrimethamine in drinking water as described in [72]. Resulting transfectants were analysed by PCR for 5’ and 3’ integration (using primers in S2 Table), cloned by limiting dilution and the absence/disruption of open reading frame in the mutants confirmed by PCR.

### Asexual growth competition assay

Equal numbers of mutant parasites (10^6^ cells) made in RMgm-7 background expressing GFP constitutively under *eef1a* promoter were mixed with wild type parasites (10^6^ cells) (RMgm- 86) expressing RFP under the same promoter and the mixture was injected into a mouse. The population of infected erythrocytes (iRBCs) was monitored by Hoechst staining and the proportion of iRBCs expressing GFP and RFP was recorded by FACS analysis over the course of seven generations. Infected blood from the first mouse was sequentially passaged into two to three mice to avoid multiple infections over this period.

### Gametocyte conversion monitoring by FACS and Giemsa stained smears during blood stage growth

Mutants made in the RMgm-164 background which expresses GFP in male gametocytes and RFP in female gametocytes along with wild type were grown in mice and peripheral blood was monitored by FACS analysis by checking for infected erythrocytes (iRBCs) by Hoechst staining and the proportion of iRBCs expressing GFP and RFP, indicative of the presence of male and female gametocytes. Mutants made in RMgm-7 background or ANKA cl15cy1 were stained with Giemsa and mature male and female gametocytes were quantified by manual observation and counting a minimum of 100 iRBCs.

### Exflagellation assay

#### Haemocytometer-based

During gametogenesis, male gametocytes undergo rapid endomitosis and DNA content is increased from 1 N to 8 N within 8 min after activation and adherent clumps of erythrocytes are formed around the activating gametocytes called exflagellation centres which were counted on haemocytometer. Mature gametocytes were obtained by treating infected mice with 25 mg/mL sulfadiazine in drinking water for 48 h before harvesting and activated in ookinete culture media (see below).

#### Flow cytometry based

Microgametogenesis was also monitored using flow cytometry when a large number of gametocytes (minimum 500,000) were monitored for activation. To do this, *P*. *berghei* parasites from the RMgm-164 background were used which express GFP in male gametocytes. iRBC from sulfadiazine treated mice were co-stained with Hoechst and Pe-CY7-ter119 (erythrocyte surface marker) and those negative for the latter, i.e. outwith an RBC membrane, were counted as activated.

### Ookinete cultures of *P*. *berghei* and conversions

Mice infected with *P*. *berghei* were given sulfadiazine in drinking water which killed all asexual stage parasites in 48 h and circulating gametocytes remain in blood. Mice were bled and infected blood was collected in unlabelled RPMI1640 containing 5 g/L Albumax II and 100 μM xanthurenic acid to activate gametocytes. Cultures were incubated at 21°C for 21 h and Giemsa stained smears were made for counting mature ookinetes and female gametes whose cumulative ratio to female gametes only gave the ookinete conversion rate.

### *in vitro* sexual crosses

Equal numbers of gametocytes from two *P*. *berghei* lines obtained from infected TO mice treated with sulfadiazine in drinking water were taken and mixed in activation media. The suspension was then incubated at 21°C for 21 h and Giemsa smears were made for counting mature ookinetes and female gametes.

### Ookinete motility assays

Ookinetes were embedded in Matrigel™ (BD Biosciences) and allowed to set for 1 h at 21°C. Fluorescent ookinetes were observed on a Leica M205 FA Fluorescence Stereomicroscope for 10.5 min and snapshots acquired every 10 sec. Tracks were constructed using Fiji software.

### Investigation of effect of 2DG on microgametogenesis and ookinete development

*P*. *berghei* parasites were pre-incubated in BSA enriched PBS with the addition of 2-deoxyglucose (2DG) at indicated concentrations at 37°C for 30 min. Parasites were then activated by replacing the media with activation media at 21°C containing the same concentration of 2DG and activation centres were counted on a haemocytometer after 30 min. Parasites were observed at later time points to confirm that there was no delayed activation. Female gametocytes emerged in the presence of 2-deoxyglucose were counted under fluorescent microscope after counterstaining with a fluorescent conjugated red blood cell marker Ter119. At least 50 female gametocytes were counted per experiment. To observe the effect of 2DG on ookinete development, gametocytes were activated then 2-deoxyglucose (25 mM) was added 30 min post activation (post). Alternatively parasites were incubated in BSA enriched PBS containing 25 mM 2-deoxyglucose for 30 min before activation in media also containing 2-deoxyglucose (pre). Conversion rates were calculated as the number of ookinetes as a percentage of the total ookinetes and unconverted females gametocyte.

### Mosquito transmission experiments

*P*. *berghei* infected mice with a parasitaemia of 5–10% were used to blood-feed a cage of 250 mosquitoes for 10 min. Mature oocysts were counted in mosquito midguts between days 8–14 using a Leica M205 FA Fluorescence Stereomicroscope. Salivary gland sporozoites were checked between days 21–25. Infected mosquitoes were allowed to feed on naïve mice for 10 min between days 21–25 and these mice were observed for parasites by making Giemsa stained blood smears between days 3–14 to check for successful transmission.

### Stable isotope labelled *P*. *berghei* cultures

Asexual and sexual stage parasites were cultured as described above in minimal media prepared based on known nutritional requirement of *Plasmodium* parasites in *in vitro* cultures [73–76] (S1 Table) lacking the unlabelled equivalent of the following labelled carbon sources, ^13^C U-glucose or ^13^C^15^N U-glutamine (Cambridge Isotope Laboratories) which were included at 2000 mg/L and 300 mg/L respectively. Briefly, for asexual cultures, parasites from the line 820m9w21dm1cl1 (gametocyte non-Producer) [27] were used to grow a synchronous infection in mice (pre-treated with phenylhydrazine-HCl 5 days before to induce reticulocytosis) with 10% parasitaemia at ring stage. Mice were bled and leucocytes were removed and each replicate containing 10^8^ cells was incubated in 12 mL media containing unlabelled or the indicated labelled carbon source. The flasks were gassed and kept at 37°C with slight shaking at 35 rpm. Each flask was harvested to collect samples for 0 h, 6 h, 12 h, 18 h, and 24 h time points (the first time point was collected after allowing for cells to equilibrate for 2 h). Uninfected mice treated similarly were also bled at the same time and samples were processed exactly as for infected blood. For gametocyte cultures, parasites from line 820em1dcl2TBB (RMgm-164, the parent producer line) [27] were grown in mice and the mice were given sulfadiazine in drinking water (30 mg/L) when the parasitaemia reached ~30% for 48 h which led to killing of all asexual stage parasites and left mature circulating gametocytes. Mice were bled and parasites were harvested as described above. Gametocytes were magnetically purified and incubated for 2 h in labelled media at 37°C before activation to allow for metabolic equilibration and were then transferred to activation media at 21°C containing labelled carbon sources, harvested at 10 min, 20 min and 30 min time points and used for quenching metabolism and metabolite extraction (as the activation process is quick, cells were purified first then activated and quenched). Ookinete cultures were performed in activation media at 21°C containing labelled carbon sources, harvested at 10 h and 21 h, magnetically purified at 21°C and used for quenching metabolism and metabolite extraction.

### Metabolite Extraction, drying and storage

At each time point, flasks were immersed in a dry ice-ethanol bath to rapidly quench metabolism. The temperature of the cell suspension was monitored using a thermometer and the flasks were removed when the temperature decreased to 8°C. The culture suspension (12 mL) was then spun at 1300 × *g* at 4°C and supernatant was removed. The cells were washed once in 500 μL cold PBS and spun again at 1300 × *g* at 4°C and supernatant was removed. The pellet was resuspended in 150 μL extraction solvent (chloroform: methanol: water: 1:3:1 v/v) with dispersion of the pellet by pipetting and mixed vigorously on shaker in cold room for 1 h. The suspensions were then sonicated for 10 min in ice-cold water bath and then centrifuged for 5 min at 15,300 × *g* at 4°C. 50 μL of the supernatant was then put into glass vials, dried under nitrogen flow at room temperature and stored at -80°C before analysis.

### Targeted GC-MS analysis

Extracts were reconstituted with extraction solvent containing an internal standard (*scyllo*-Inositol, SI, 1 nmol) and dried in vacuum. Then 20 μL of 30 mg/mL methoxyamine-HCl in pyridine was added to the vials, mixed and incubated overnight at 30°C. This was followed by addition of 20 μL of BSTFA + 1% TMCS Silylation reagent, mixing and overnight incubation at 37°C. 1 μL of the reconstituted and derivatised sample mix was injected onto an Agilent 7890A-5975C GC-MS instrument, equipped with a VF5-MS column (30 m, 0.25 mm inner diameter) with helium as the carrier gas. The oven temperature was held at 70°C (1 min), then ramped at 1°C/min to 76°C, then 5°C/min to 325°C and held for 10 min. As a control, a mixture of glycolytic and TCA cycle standards were also derivatized and run with the samples to serve as authentic reference standards for retention time and spectra. Data analysis was performed manually on the Agilent ChemStation platform and metabolites were identified based on their GC retention time and mass spectra compared to authentic reference standards. The level of labelling was estimated as the percent of metabolite pool containing one or more labelled ^13^C atoms and the distribution of mass isotopologues of individual metabolites was corrected for naturally abundant isotopes in both the metabolite and derivatizing agent as described previously [10,77]. All stable isotope labelling data is available to download as a summary table in MS-Excel and raw MS files from the Figshare repository accessible from https://figshare.com/s/aabe6ed0eba25acddcc1.

## Supporting Information

S1 Fig Flow of carbon skeletons through the glycolytic and TCA cycle pathways. With U-^13^C-glucose labelling, provided the classical glycolysis to TCA cycle pathways operate in the canonical manner, starting from a fully labelled (^13^C_6_) glucose molecule, all glycolytic intermediates show +6 or +3 C labelling- panel (A) and all TCA cycle metabolites should show +2, +4 or +6 labelling (A, B and C). The expected abundance of +2 to +6 labelling will be in decreasing order as the carbon skeletons have to go round the TCA cycle three times to achieve maximal (+6) labelling. Anaplerotic reactions undergoing intermediary carbon metabolism in the cytosol will give rise to +3 labelled intermediates (D). With U-^13^C^15^N-glutamine as the labelled carbon source, as glutamine interconverts with the TCA cycle intermediate alpha-ketoglutarate, glycolytic metabolites will show no labelling and if the canonical TCA cycle is operative, TCA intermediates will show +4 labelling (E). In case of reductive carboxylation of alpha-ketoglutarate, it is also possible to see +5 labelling of citrate (F).
(TIF)

S2 Fig Generation of *P*. *berghei* metabolic mutants. Top right panel: schematic representation of gene deletion strategy. HA1, Homologous Arm 1; HA2, Homologous Arm 2; GOI, Gene of Interest; SM, Selectable Marker. Left and bottom right panels: Gel electrophoresis of indicated PCR products to confirm integration of selection cassette, disruption of genes and clonality of mutant parasites. Appearance of bands in the wt panel for *gdh2*^*-*^ (G1105 and G1106) and *put trp*^*-*^ (G1010 and G1016) correspond to the predicted size of the wt locus indicating the presence of wt population and non-clonality. Appearance of bands in the wt panel in *gdh3*^*-*^ (G1012cl1) and *oat*^*-*^ (G1009cl1 and G1015cl2) is due to unspecific activity of primers and does not correspond to the predicted size of wt locus which is 1.2 kb and 1.1 kb, respectively. Lines G1141cl1, G1007cl1, G1105, G1006cl3, G1126cl1, G1009cl1, G1103cl1, G662cl3, G663cl2, G664cl2, G863cl2 and G1010 were generated in parent line RMgm-7 which expresses GFP constitutively under *eef1a* promoter. Lines G1140cl1, G1013cl1, G1106, G1012cl1, G1015cl2, G1104cl1 and G1016 were generated in parent line RMgm-164 which expresses GFP in male gametocytes (under dynein heavy chain promoter) and RFP in female gametocytes (under LCCL domain-containing protein CCP2 promoter). Line G1124cl1 was generated in parent line ANKA cl15cy1 and expresses GFP constitutively under *hsp70* promoter.
(TIF)

S3 Fig Time taken for asexual parasites to grow to mature schizont stage. Coloured lines indicate non-linear fit of percentage of mature schizonts observed in *in vitro* synchronous cultures of wt and mutant *P*. *berghei* parasites 22 h post-invasion. Data representative of n = 2 independent biological replicates. P-value ***p < 0.001, Repeated Measures ANOVA- Dunnett's Test with wt control.
(TIF)

S4 Fig Mosquito stage development of *P*. *berghei* metabolic mutants in mid-gut. Mosquito mid guts showing mature oocysts at day 14 post-infection in *P*. *berghei* mutant parasite infected mosquitoes.
(TIF)

S5 Fig Mosquito stage development of *P*. *berghei* metabolic mutants in salivary glands. Mosquito salivary glands showing sporozoites at day 21 in *P*. *berghei* mutant parasite infected mosquitoes.
(TIF)

S6 Fig *P*. *berghei in vitro* growth comparison between minimal media and RPMI 1640. *P*. *berghei in vitro* growth in minimal media + Albumax normalised to growth in RPMI 1640 + Albumax. Dotted line represents observations for RPMI 1640 + Albumax. Error bars indicate SD of n = 3 biological replicates.
(TIF)

S7 Fig Detailed kinetics of *in vivo* growth assay presented in Fig 2B. Each mutant (expressing GFP) was mixed with an equal number of wt parasites (parental expressing RFP) and the mixture was passaged into mice to avoid multiple infectivity in RBCs. Arrows indicate subsequent passages into new mice on days 3 and 6 post initial passage. The ratio of RFP to GFP which was 50% (normalised to control) on day 1 was monitored over 7 generations over as many days. Error bars indicate SD of n = 3 biological replicates.
(TIF)

S1 Table *P*. *berghei in vitro* growth minimal media composition. (DOCX)

S2 Table List of primers. (DOCX)

S3 Table Phenotypic summary of all the metabolic mutants generated in this study. (DOCX)
