# Supplementary figures and images for: Stage-Specific Changes in Plasmodium Metabolism Required for Differentiation and Adaptation to Different Host and Vector Environments
Source: PLoS Pathog. 2016 Dec 27;12(12):e1006094. doi: 10.1371/journal.ppat.1006094 (PMC5189940; doi:10.1371/journal.ppat.1006094)

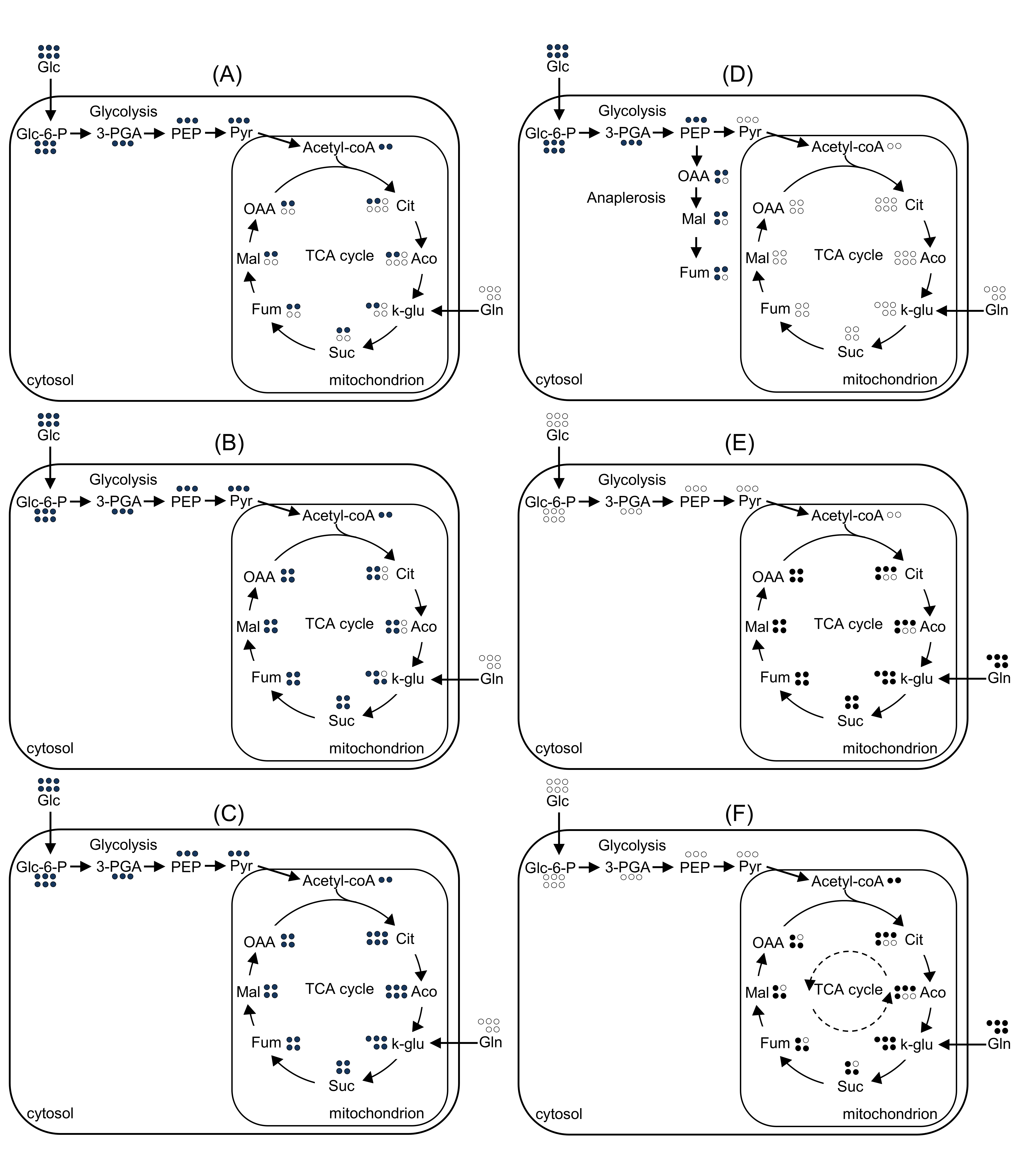

Supplement: S1 Fig — With U-13C-glucose labelling, provided the classical glycolysis to TCA cycle pathways operate in the canonical manner, starting from a fully labelled (13C6) glucose molecule, all glycolytic intermediates show +6 or +3 C labelling- panel (A) and all TCA cycle metabolites should show +2, +4 or +6 labelling (A, B and C). The expected abundance of +2 to +6 labelling will be in decreasing order as the carbon skeletons have to go round the TCA cycle three times to achieve maximal (+6) labelling. Anaplerotic reactions undergoing intermediary carbon metabolism in the cytosol will give rise to +3 labelled intermediates (D). With U-13C15N-glutamine as the labelled carbon source, as glutamine interconverts with the TCA cycle intermediate alpha-ketoglutarate, glycolytic metabolites will show no labelling and if the canonical TCA cycle is operative, TCA intermediates will show +4 labelling (E). In case of reductive carboxylation of alpha-ketoglutarate, it is also possible to see +5 labelling of citrate (F). (TIF) [file ppat.1006094.s001.tif]

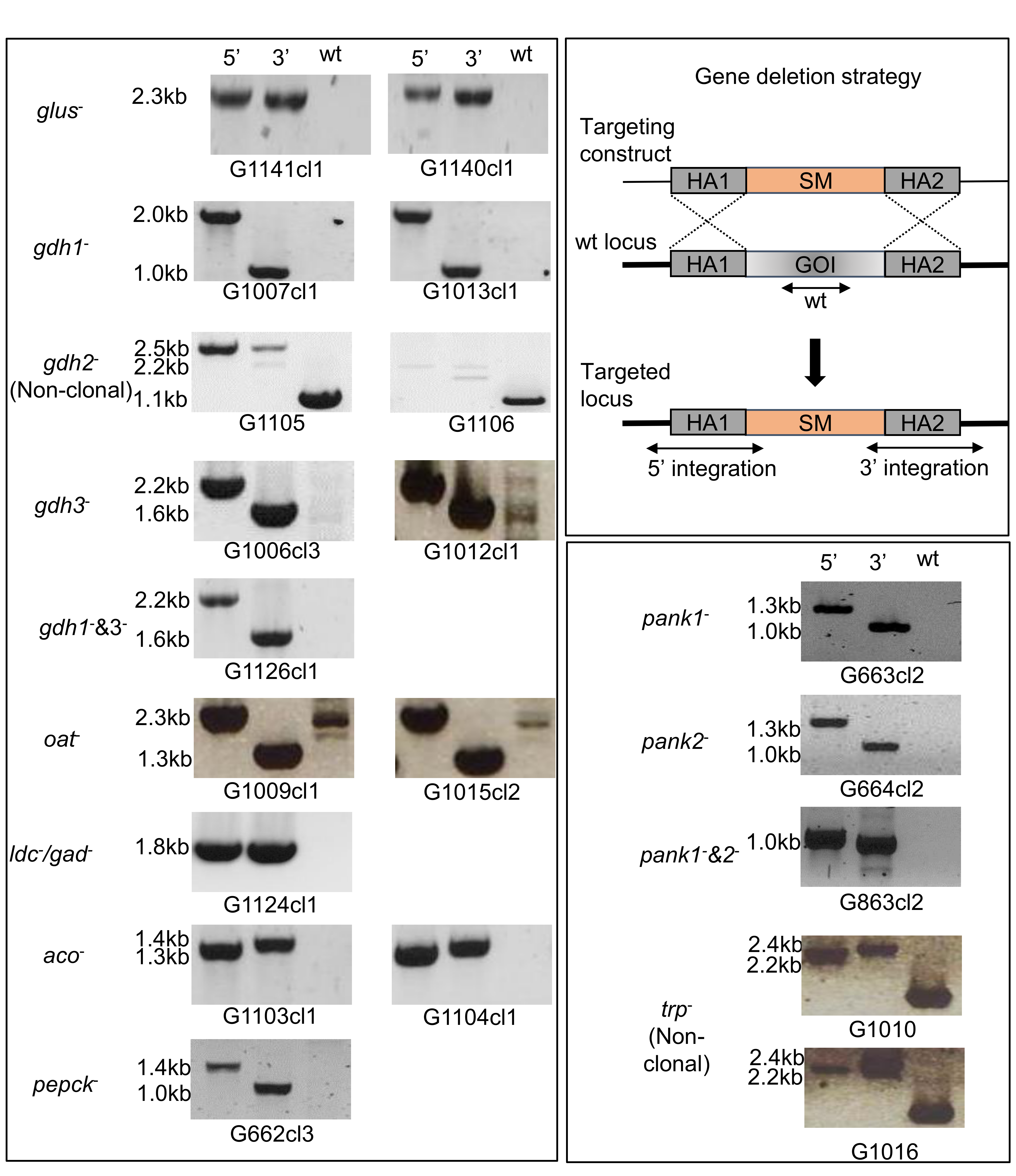

Supplement: S2 Fig — Top right panel: schematic representation of gene deletion strategy. HA1, Homologous Arm 1; HA2, Homologous Arm 2; GOI, Gene of Interest; SM, Selectable Marker. Left and bottom right panels: Gel electrophoresis of indicated PCR products to confirm integration of selection cassette, disruption of genes and clonality of mutant parasites. Appearance of bands in the wt panel for gdh2- (G1105 and G1106) and put trp- (G1010 and G1016) correspond to the predicted size of the wt locus indicating the presence of wt population and non-clonality. Appearance of bands in the wt panel in gdh3- (G1012cl1) and oat- (G1009cl1 and G1015cl2) is due to unspecific activity of primers and does not correspond to the predicted size of wt locus which is 1.2 kb and 1.1 kb, respectively. Lines G1141cl1, G1007cl1, G1105, G1006cl3, G1126cl1, G1009cl1, G1103cl1, G662cl3, G663cl2, G664cl2, G863cl2 and G1010 were generated in parent line RMgm-7 which expresses GFP constitutively under eef1a promoter. Lines G1140cl1, G1013cl1, G1106, G1012cl1, G1015cl2, G1104cl1 and G1016 were generated in parent line RMgm-164 which expresses GFP in male gametocytes (under dynein heavy chain promoter) and RFP in female gametocytes (under LCCL domain-containing protein CCP2 promoter). Line G1124cl1 was generated in parent line ANKA cl15cy1 and expresses GFP constitutively under hsp70 promoter. (TIF) [file ppat.1006094.s002.tif]

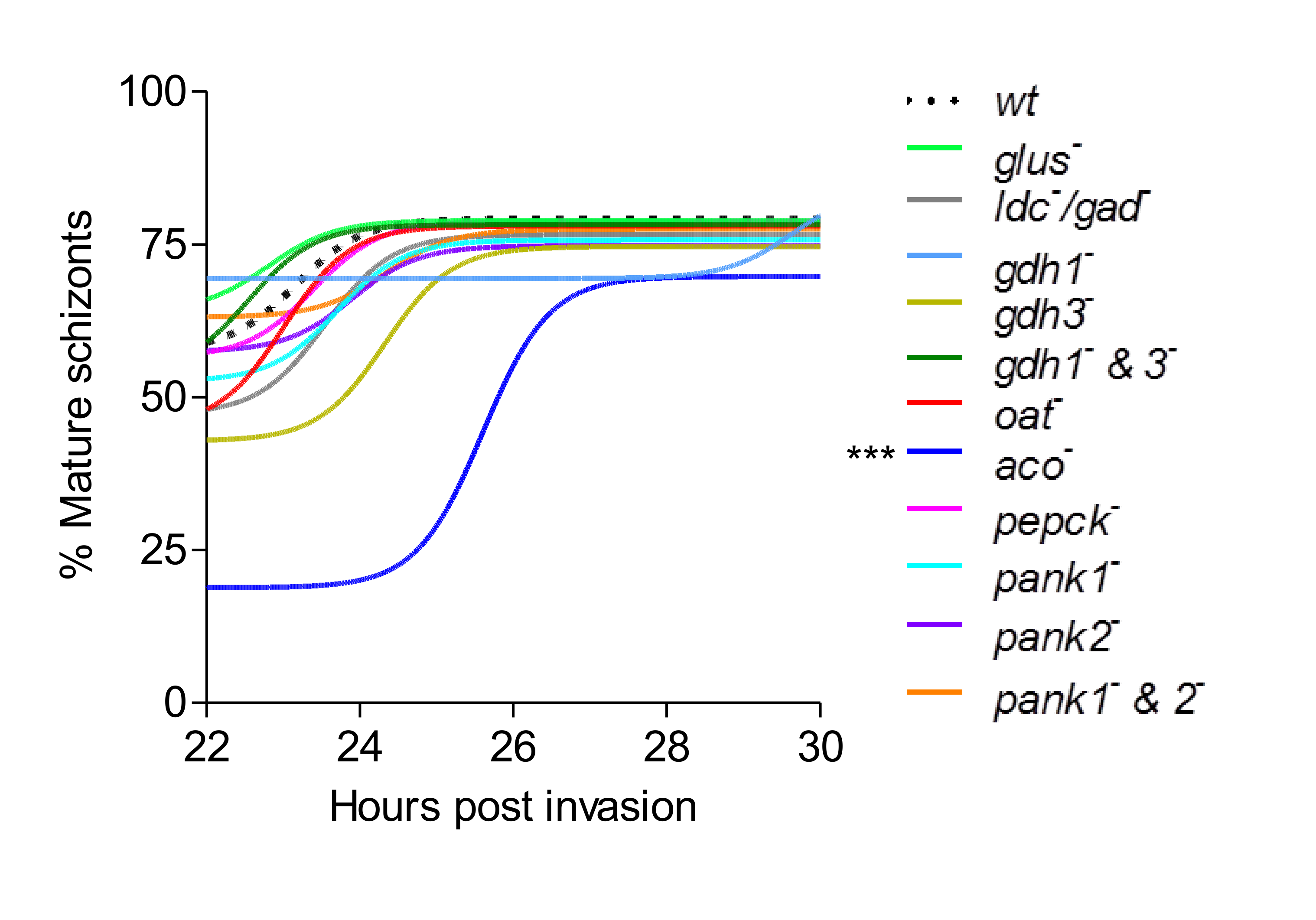

Supplement: S3 Fig — Coloured lines indicate non-linear fit of percentage of mature schizonts observed in in vitro synchronous cultures of wt and mutant P. berghei parasites 22 h post-invasion. Data representative of n = 2 independent biological replicates. P-value ***p < 0.001, Repeated Measures ANOVA- Dunnett's Test with wt control. (TIF) [file ppat.1006094.s003.tif]

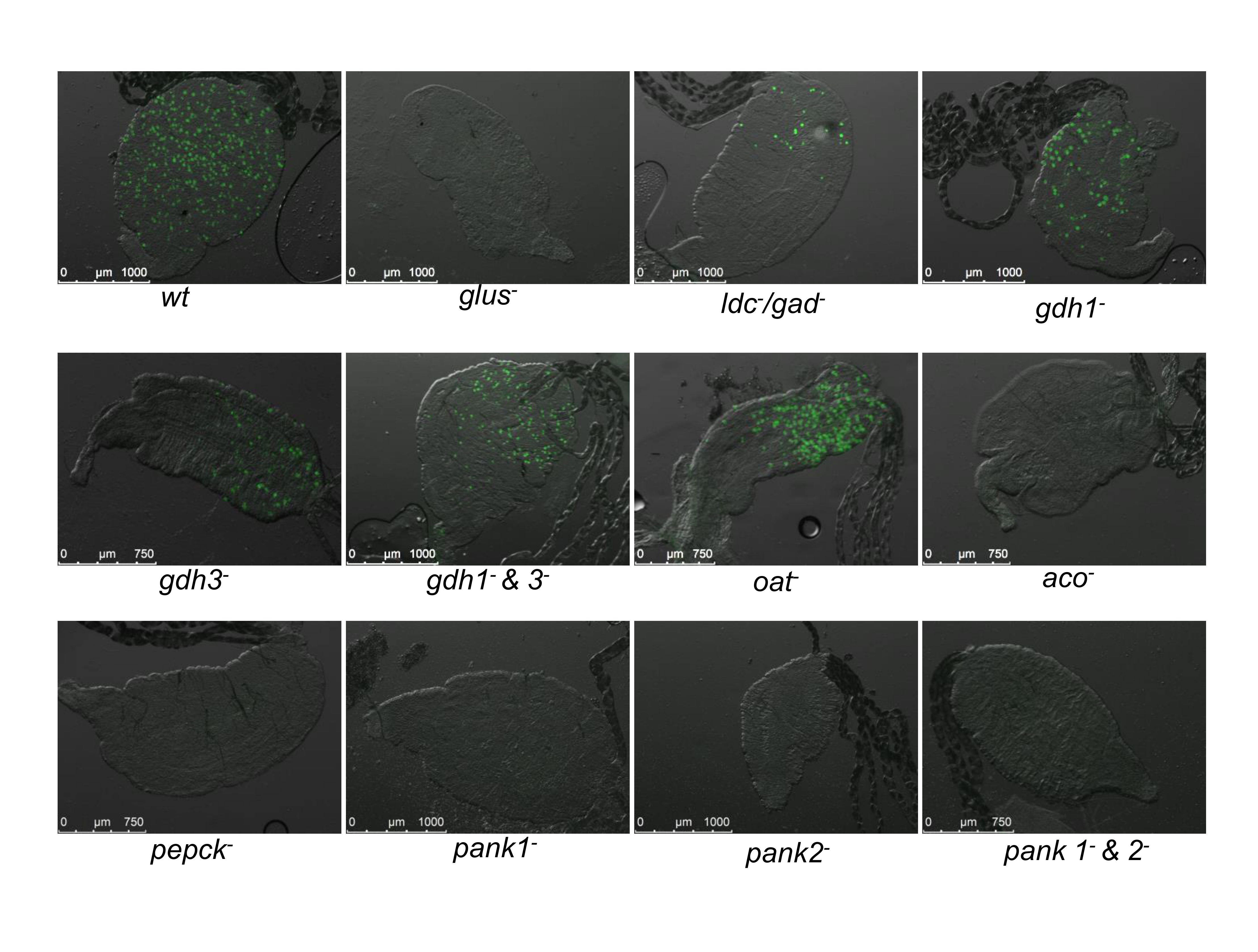

Supplement: S4 Fig — Mosquito mid guts showing mature oocysts at day 14 post-infection in P. berghei mutant parasite infected mosquitoes. (TIF) [file ppat.1006094.s004.tif]

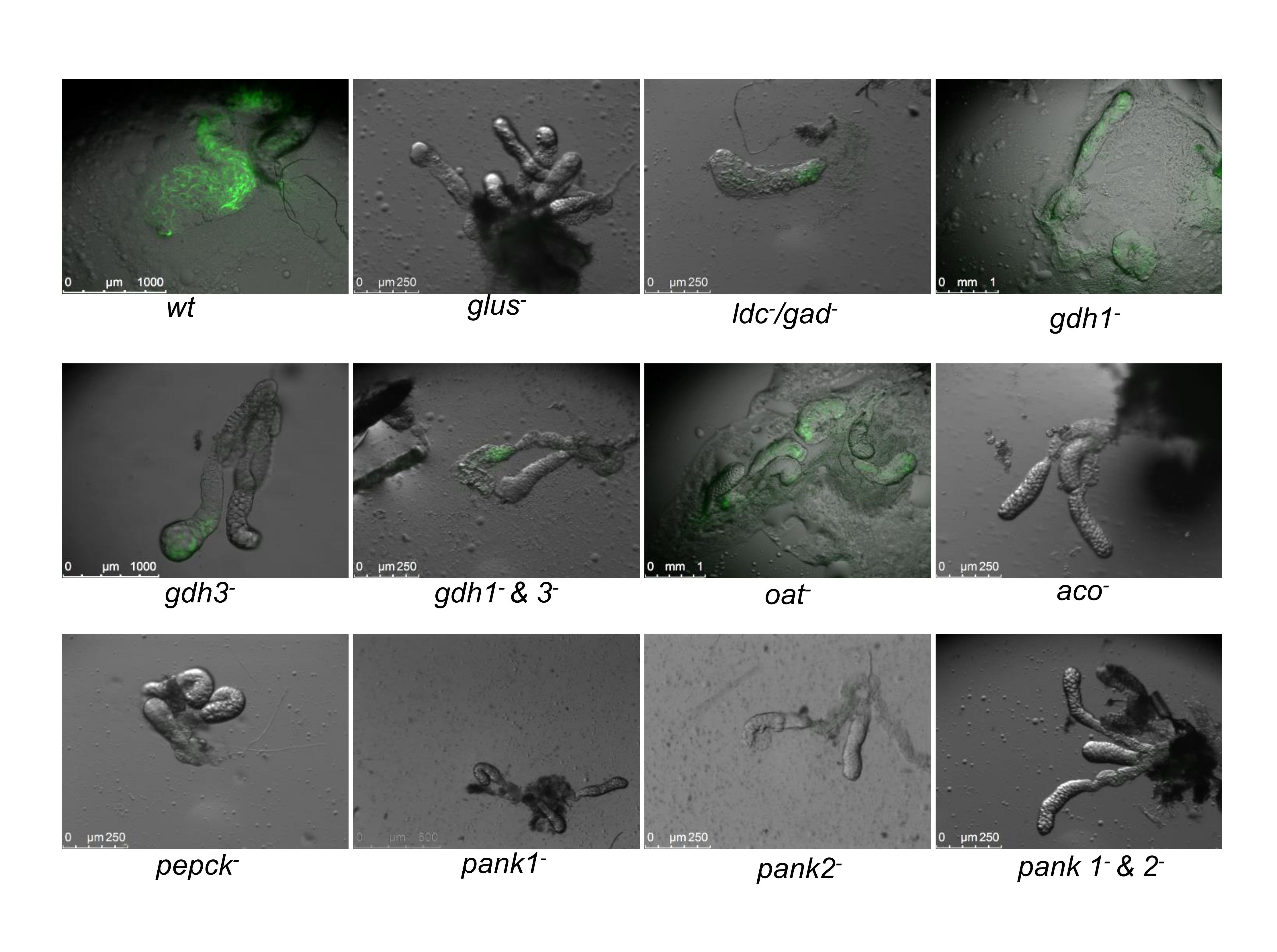

Supplement: S5 Fig — Mosquito salivary glands showing sporozoites at day 21 in P. berghei mutant parasite infected mosquitoes. (TIF) [file ppat.1006094.s005.tif]

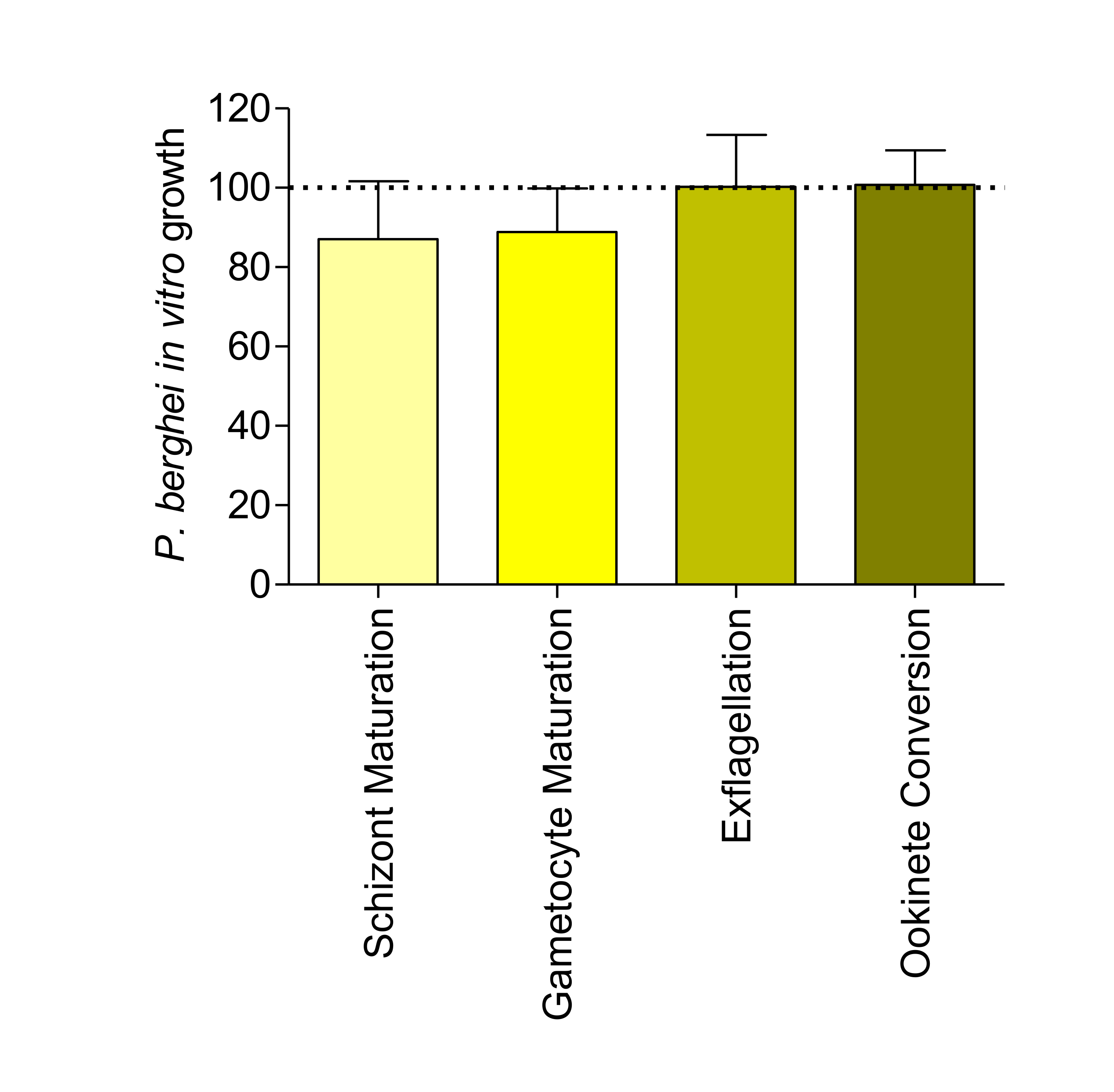

Supplement: S6 Fig — P. berghei in vitro growth in minimal media + Albumax normalised to growth in RPMI 1640 + Albumax. Dotted line represents observations for RPMI 1640 + Albumax. Error bars indicate SD of n = 3 biological replicates. (TIF) [file ppat.1006094.s006.tif]

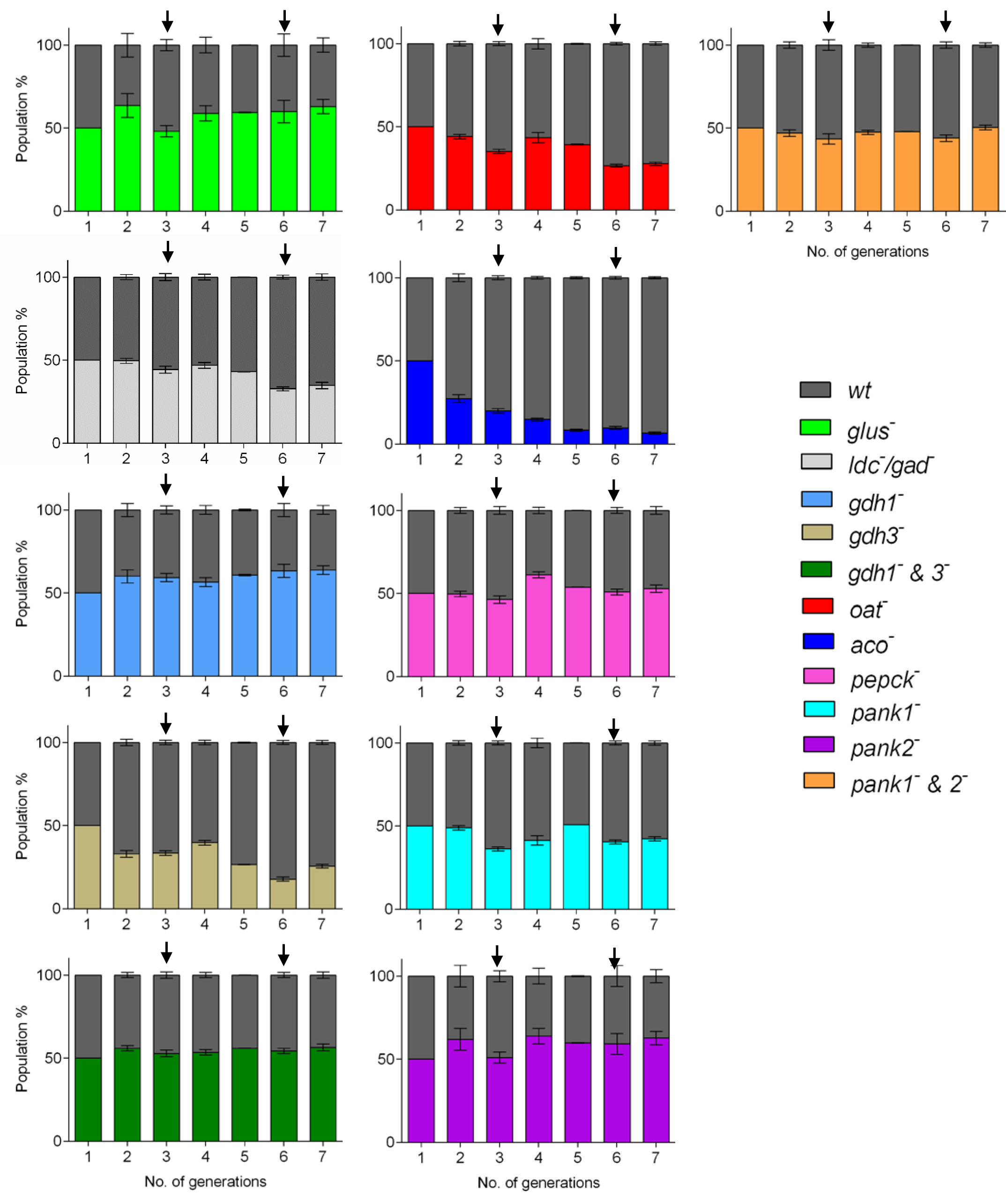

Supplement: S7 Fig — Each mutant (expressing GFP) was mixed with an equal number of wt parasites (parental expressing RFP) and the mixture was passaged into mice to avoid multiple infectivity in RBCs. Arrows indicate subsequent passages into new mice on days 3 and 6 post initial passage. The ratio of RFP to GFP which was 50% (normalised to control) on day 1 was monitored over 7 generations over as many days. Error bars indicate SD of n = 3 biological replicates. (TIF) [file ppat.1006094.s007.tif]
