# Supplementary material for: Stage-Specific Changes in Plasmodium Metabolism Required for Differentiation and Adaptation to Different Host and Vector Environments
Source: PLoS Pathog. 2016 Dec 27;12(12):e1006094. doi: 10.1371/journal.ppat.1006094 (PMC5189940; doi:10.1371/journal.ppat.1006094)
Supplement: S1 Table — (DOCX) [file ppat.1006094.s008.docx]

# S1 Table

***P. berghei* *in vitro* growth minimal media composition.** All components were mixed to 1x concentration (as shown in S1 table below) and Pen-Strep antibiotic mix (1 ml containing 10,000 units penicillin and 10 mg streptomycin per litre) was added just before use. pH was adjusted to 7.3 and complete media was then sterile filtered using a 0.22µ filter. When a labelled carbon source was to be used for cultures (e.g. U-^13^C-Glucose or U-^13^C^15^N-Glutamine), the corresponding component was replaced with the isotopically labelled form. For gametocyte activation and ookinete cultures, Xanthurenic acid was added to media at a final concentration of 100µM before filtration.

| Components | mg/L (final concentration 1x) |
| --- | --- |
| Ca(NO_3_)_2_.4H_2_0 | 100 |
| KCl | 400 |
| MgSO_4_ (anhydrous) | 48.8 |
| NaCl | 5300 |
| NaHCO_3_ | 2000 |
| Na_2_HPO_4_ (anhydrous) | 800 |
| Hypoxanthine | 4.1 |
| D-Glucose | 2000 |
| Glutathione | 1 |
| HEPES | 5958 |
| Phenol Red | 5 |
| L-Cystine.2HCl | 65 |
| L-Glutamic Acid | 20 |
| L-Glutamine | 300 |
| L-Isoleucine | 50 |
| L-Methionine | 15 |
| L-Proline | 20 |
| L-Tyrosine.2Na.2H_2_O | 29 |
| Ca-Pantothenate | 0.25 |
| AlbuMAX® | 5000 |
