# Supplementary material for: Stage-Specific Changes in Plasmodium Metabolism Required for Differentiation and Adaptation to Different Host and Vector Environments
Source: PLoS Pathog. 2016 Dec 27;12(12):e1006094. doi: 10.1371/journal.ppat.1006094 (PMC5189940; doi:10.1371/journal.ppat.1006094)
Supplement: S2 Table — (DOCX) [file ppat.1006094.s009.docx]

# S2 Table

**List of primers.** Primers used for PCR amplification for creating gene disruption/ deletion mutants and confirming integration at the targeted locus.

| Primer | Sequence | Description |
| --- | --- | --- |
| GU3252 | ACTGGGCCCGAGTATAATAGCATATATATCCATGTATTAATTGCG | *gluS* 5’homology arm forward |
| GU3253 | GTACCGCGGGGCTATCGTATAATCCATGTTTGGCCG | *gluS* 5’ homology arm reverse |
| GU3254 | ACTGGTACCCCCTGGTTGGAAATATTATACCGAAGAGC | *gluS* 3’homology arm forward |
| GU3255 | GTACTCGAGGAGCGATTTAAGATGTACATCTAGTAAATATTGCTC | *gluS* 3’ homology arm reverse |
| GU3260 | CATGTGTATAATAAGAGGAATGC | *gluS* disruption 5’ integration forward |
| GU0205 | CTATTTATGAATCATTGAAGAGAC | *gluS* disruption 5’ integration reverse in plasmid |
| GU3261 | CACAATAAATCATTATAATCAGCG | *gluS* disruption 3’ integration reverse |
| GU0204 | GTCTCTTCAATGATTCATAAATAG | *gluS* disruption 3’ integration forward in plasmid |
| GU3266 | CATTTAACATAATCATATCTCCTATGG | *gluS* wt locus forward |
| GU3267 | CTAAATATTCTCTAACTTCGTTTGCC | *gluS* wt locus reverse |
| GU2943 | ACTAAGCTTCTATGTATATATTTTTCGACATTTAACTGC | *gdh1* 5’homology arm forward |
| GU2944 | CATCCGCGGCAATGTAACAAATTATATAAGTATTTATTTGTTTGTAT | *gdh1* 5’ homology arm reverse |
| GU2945 | TCAGGTACCCGAGGATGCTTCGTATGATCTTAGCCAC | *gdh1* 3’homology arm forward |
| GU2946 | CTACTCGAGCCGAGATGTTGTAGAGCATCATATGTATGAC | *gdh1* 3’ homology arm reverse |
| GU3009 | GTATGTGTGCATATATGCTTATTCACCA | *gdh1* ko 5’ integration forward |
| GU0205 | CTATTTATGAATCATTGAAGAGAC | *gdh1* ko 5’ integration reverse in plasmid |
| GU3010 | TTGGGAACATATTGTCTTTACAAGAGATG | *gdh1* ko 3’ integration reverse |
| GU0204 | GTCTCTTCAATGATTCATAAATAG | *gdh1* ko 3’ integration forward in plasmid |
| GU3012 | AAATGACGCAATGCCGAGATAGTACCG | *gdh1* wt locus forward |
| GU3011 | CTATTGTTGATGGCATATTAGCACCTTCAC | *gdh1* wt locus reverse |
| GU2947 | ATCGGGCCCGAATATCTTATTTTGTATGTGCATAATATGTGTC | *gdh2* 5’homology arm forward |
| GU2948 | CTACCGCGGGGGGGTTTATAGTTCCATTGGGTAT | *gdh2* 5’ homology arm reverse |
| GU2949 | TCAGGTACCCAAATGGAACCGAACCCTCAATTATATTTCC | *gdh2* 3’homology arm forward |
| GU2950 | CTACTCGAGCACAATGAACATATGTATACCGATCTATTTGG | *gdh2* 3’ homology arm reverse |
| GU3084 | CAGAAATGTGTGGAACCTTACATGG | *gdh2* ko 5’ integration forward |
| GU0205 | CTATTTATGAATCATTGAAGAGAC | *gdh2* ko 5’ integration reverse in plasmid |
| GU3085 | GGCTCAAATTTCACATCGCCTGAAG | *gdh2* ko 3’ integration reverse |
| GU0204 | GTCTCTTCAATGATTCATAAATAG | *gdh2* ko 3’ integration forward in plasmid |
| GU3007 | AAATATTGTGCAACATTGCCACTTCCACTA | *gdh2* wt locus forward |
| GU3008 | GATGATATTTTGTCACAACTCAATGTTAAGTG | *gdh2* wt locus reverse |
| GU2951 | ACTAAGCTTGGTTATGCAATATACTGACAAATATGTATAGCC | *gdh3* 5’homology arm forward |
| GU2952 | CATCCGCGGTAAGGGCGGGGGAGTAATATATATGTAATGT | *gdh3* 5’ homology arm reverse |
| GU2953 | TCAGGTACCGAGTGTGCATGTTTTACTACATGTTTTGTATTAC | *gdh3* 3’homology arm forward |
| GU2954 | CTACTCGAGGGGAAATCAATCACTTCCGTTATACTACAA | *gdh3* 3’ homology arm reverse |
| GU3013 | GTTCAATTACTTTCTTATTCTGTGAAAACAACC | *gdh3* ko 5’ integration forward |
| GU0205 | CTATTTATGAATCATTGAAGAGAC | *gdh3* ko 5’ integration reverse in plasmid |
| GU3014 | CAAGAACGAAACATGTATAAGAAGCGAAC | *gdh3* ko 3’ integration reverse |
| GU0204 | GTCTCTTCAATGATTCATAAATAG | *gdh3* ko 3’ integration forward in plasmid |
| GU3015 | ATGTCCATCCGTAACATTATCACCTGCA | *gdh3* wt locus forward |
| GU3016 | CATTATTCCAGTCCCTAAATTACTAGATCCAG | *gdh3* wt locus reverse |
| GU2955 | ATCGGGCCCGCGTAAGGCATGTATACATGATGTTATGTA | *oat* 5’homology arm forward |
| GU2956 | CTACCGCGGAGTACAAATGCAGGACAGAACACATTGCAA | *oat* 5’ homology arm reverse |
| GU2957 | TCAGGTACCTAGAGGCATGTTGATGTTTACTAGTATATGC | *oat* 3’homology arm forward |
| GU2958 | CTACTCGAGATATGTCGTTTGTGTTAAGAATGATATGTACTG | *oat* 3’ homology arm reverse |
| GU3017 | CACAAGCAAAACGATACACATTCACTTGTT | *oat* ko 5’ integration forward |
| GU0205 | CTATTTATGAATCATTGAAGAGAC | *oat* ko 5’ integration reverse in plasmid |
| GU3018 | GTAATAATAAATGTACTAATATCTTCACCGTC | *oat* ko 3’ integration reverse |
| GU0204 | GTCTCTTCAATGATTCATAAATAG | *oat* ko 3’ integration forward in plasmid |
| GU3019 | GGAGCTCACAATTATGATCCCATTCCC | *oat* wt locus forward |
| GU3020 | GTTTTGTCATGAACATCCCTAGTAATTAAACC | *oat* wt locus reverse |
| GU3211 | ACTCCGCGGCGTTGATATTGAATAAGCAAGTGCACGC | *ldc/gad* 5’homology arm forward |
| GU3212 | GTAGCGGCCGCCATGTAAATCGCTATGATCATCATGTGTTG | *ldc/gad* 5’ homology arm reverse |
| GU3213 | ACTCTCGAGGTATAGTTTGTTCTGATGCGAATAATGGAACAG | *ldc/gad* 3’homology arm forward |
| GU3214 | GTAGGTACCGAATCTGTGCGATTACCACTTGAATAATTATTTGG | *ldc/gad* 3’ homology arm reverse |
| GU3511 | GGAAGCGTATAATTTCCACATATC | *ldc/gad* disruption 5’ integration forward |
| GU3512 | GCTCACCAACAAAATATTACAAATC | *ldc/gad* disruption 5’ integration reverse in plasmid |
| GU3258 | CACATCCAAATTTCCATTTACCACATGC | *ldc/gad* disruption 3’ integration forward in plasmid |
| GU3259 | GATCTGCATTATTTTCATCATTCTCTTCAATC | *ldc/gad* disruption 3’ integration reverse |
| GU3538 | CCTATAGGTGTATTTCACGCATTAG | *ldc/gad* wt locus forward |
| GU3539 | ACTCTTCATACAATCTACGCATACAC | *ldc/gad* wt locus reverse |
| GU2767 | CCGAAGCTTCGAGGGATTAGTAATTTATTATATTATATATGTG | *aco* 5’homology arm forward |
| GU2768 | ATCCGCGGCACCGAAACTTTTATGATATATTTTGTGTAAG | *aco* 5’ homology arm reverse |
| GU2769 | CCGGGTACCCTTATGAAGAAGATATGGATAAAATATTTCTTC | *aco* 3’homology arm forward |
| GU2770 | ATCTCGAGCCCTCAATTTAGACAATAATTGTGATAG | *aco* 3’ homology arm reverse |
| GU2826 | CAATCCGGGCAGTATTGTATATAGTAAAG | *aco* ko 5’ integration forward |
| GU2061 | GTAAACTTAAGCATAAAGAGCTCG | *aco* ko 5’ integration reverse in plasmid |
| GU2827 | GAGGAAAATATCGAATATAATAATAGTCTTCG | *aco* ko 3’ integration reverse |
| GU0204 | GTCTCTTCAATGATTCATAAATAG | *aco* ko 3’ integration forward in plasmid |
| GU2828 | GCTGGTGTGAATTTCAGTAACTATGTG | *aco* wt locus forward |
| GU2829 | CTAAAGCTGGAGAAGCTAAATAATTTGC | *aco* wt locus reverse |
| GU1967 | ATGGGCCCCCCCCTTATCTTAAACTTCGAAATGTAAAAACA | *pepck* 5’homology arm forward |
| GU1968 | CGTCCGCGGGACTTTGAATTGATTAGCTATTTTGAAATATATTTTCTTC | *pepck* 5’ homology arm reverse |
| GU1969 | CTGGTACCCAAATTCTCGGTAGATAAAATTTATATTGGGG | *pepck* 3’homology arm forward |
| GU1970 | ATCTCGAGGATCTTCATAAATTTTATAAACTCAAAAATACGACTCG | *pepck* 3’ homology arm reverse |
| GU2262 | GAGCCTATGATAAGAATATTCACTAACC | *pepck* ko 5’ integration forward |
| GU0205 | CTATTTATGAATCATTGAAGAGAC | *pepck* ko 5’ integration reverse in plasmid |
| GU2263 | GGAACATACCAAATGCGGGCAAAC | *pepck* ko 3’ integration reverse |
| GU0204 | GTCTCTTCAATGATTCATAAATAG | *pepck* ko 3’ integration forward in plasmid |
| GU2264 | GTAATCGATGAAATAAGGAGGAACTTGG | *pepck* wt locus forward |
| GU2265 | CATTCGTGTATATTTTAATGGAATTCTTTCTCC | *pepck* wt locus reverse |
| GU1867 | GCCAAGCTTGGTCAAACAGGTTTGTAAATATAAAATAAGGCTATAAGGCC | *pank1* 5’homology arm forward |
| GU1868 | ATCCGCGGGTGCAGAAATGACACTCCCCGTCTTAGATTAATATAAAC | *pank1* 5’ homology arm reverse |
| GU1869 | CTAGGTACCGCTAGTGTTTTGTAAACATGATTTTATTTTACCGCC | *pank1* 3’homology arm forward |
| GU1870 | ATCTCGAGCCCATATGTATACAATTGTTTAACGACATTGGCGC | *pank1* 3’ homology arm reverse |
| GU2254 | GTTTTACACTATTATTGTATATGCTCACGATTTC | *pank1* ko 5’ integration forward |
| GU0205 | CTATTTATGAATCATTGAAGAGAC | *pank1* ko 5’ integration reverse in plasmid |
| GU2255 | CAGTTATGCTTATCATTTTCACACTTCTTG | *pank1* ko 3’ integration reverse |
| GU0204 | GTCTCTTCAATGATTCATAAATAG | *pank1* ko 3’ integration forward in plasmid |
| GU2256 | GGAGAATAATAAAAATGTAGAAGAAAATGCTGA | *pank1* wt locus forward |
| GU2257 | CTTCCGGCAATGTATCATCCTTAC | *pank1* wt locus reverse |
| GU1871 | CCGAAGCTTGGTGGTTTCAAATATTATAAAAATAATGCACC | *pank2* 5’homology arm forward |
| GU1872 | ATCCGCGGCTTTTGTAATTTCCCCCCACAATTTGATTACCTC | *pank2* 5’ homology arm reverse |
| GU1873 | CCGGGTACCGTCAATCTGGTCACATAATATATATTGCATGTGC | *pank2* 3’homology arm forward |
| GU1874 | ATCTCGAGGTCAACATAAATAAAACTATCACCAAACGTGCGCAGTG | *pank2* 3’ homology arm reverse |
| GU2258 | GTTATATACATACAACGCGCATTAACATAG | *pank2* ko 5’ integration forward |
| GU0205 | CTATTTATGAATCATTGAAGAGAC | *pank2* ko 5’ integration reverse in plasmid |
| GU2259 | CATAAGCTTTACATTAAATGCCATGTGTG | *pank2* ko 3’ integration reverse |
| GU0204 | GTCTCTTCAATGATTCATAAATAG | *pank2* ko 3’ integration forward in plasmid |
| GU2260 | GGGAATATAATAGGAGTAGAATGCTCC | *pank2* wt locus forward |
| GU2261 | CACAATTAAGATCTTCATCGGACAACG | *pank2* wt locus reverse |
| GU2959 | ATCGGGCCCCGGAACTTTTCGAGCTATTATCATTATTTAATG | *put trp* 5’homology arm forward |
| GU2960 | CTACCGCGGTTGTAGTTGTCTTTGCTTGGGTCTATATGT | *put trp* 5’ homology arm reverse |
| GU2961 | TCAGGTACCGAGCCAGGTGTTTATGTGTGTATATAAATGAT | *put trp* 3’homology arm forward |
| GU2962 | CTACTCGAGATCAGAACTTATCTCATACATAAATCTGGTGG | *put trp* 3’ homology arm reverse |
| GU3021 | TCAATCGTTTCGTTAGCTAAATTGTCGTTTC | *put trp* ko 5’ integration forward |
| GU0205 | CTATTTATGAATCATTGAAGAGAC | *put trp* ko 5’ integration reverse in plasmid |
| GU3022 | GCTTATACAAGACTTAAGTAGTTAGACAG | *put trp* ko 3’ integration reverse |
| GU0204 | GTCTCTTCAATGATTCATAAATAG | *put trp* ko 3’ integration forward in plasmid |
| GU3023 | CTAAAATGGCAAAAACAACCCATCCATAATG | *put trp* wt locus forward |
| GU3024 | TCTTGAATTTTCAAGGAGGGATTGTTCG | *put trp* wt locus reverse |
