# Supplementary material for: Stage-Specific Changes in Plasmodium Metabolism Required for Differentiation and Adaptation to Different Host and Vector Environments
Source: PLoS Pathog. 2016 Dec 27;12(12):e1006094. doi: 10.1371/journal.ppat.1006094 (PMC5189940; doi:10.1371/journal.ppat.1006094)
Supplement: S3 Table — (DOCX) [file ppat.1006094.s010.docx]

# S3 Table

**Phenotypic summary of all the metabolic mutants generated in this study.**

| **Genes disrupted** | **Gene abbreviation** | **Protein abbreviation** | **Gene id** | **Mutant cloned?** | **Phenotype** |
| --- | --- | --- | --- | --- | --- |
| glutamate synthase | *glus* | GluS | PBANKA_1009500 | Yes | Reduced exflagellation, oocysts reduced and smaller, no sporozoites, transmission blocked |
| glutamate dehydrogenase 1 | *gdh1* | GDH1 | PBANKA_102620 | Yes | No major phenotype, mutant able to complete whole lifecycle and transmit through mosquito. |
| glutamate dehydrogenase 2 | *gdh2* | GDH2 | PBANKA_101400 | No | - |
| glutamate dehydrogenase 3 | *gdh3* | GDH3 | PBANKA_122820 | Yes | No major phenotype, mutant able to complete whole lifecycle and transmit through mosquito. |
|  | *gdh1 & 3* | GDH1 & 3 |  | Yes | Reduced exflagellation but mutant able to complete whole lifecycle and transmit through mosquito. |
| ornithine amino transferase | *oat* | OAT | PBANKA_010740 | Yes | No major phenotype, mutant able to complete whole lifecycle and transmit through mosquito. |
| lysine decarboxylase/ glutamate decarboxylase | *ldc/gad* | LDC/GAD | PBANKA_100340 | Yes | Reduced exflagellation but mutant able to complete whole lifecycle and transmit through mosquito. |
| aconitase | *aco* | ACO | PBANKA_135520 | Yes | Normal gametocytes but ookinete development severely affected. No oocysts or sporozoites, transmission blocked |
| phosphoenolpyruvate carboxykinase | *pepck* | PEPCK | PBANKA_135590 | Yes | No oocysts or sporozoites, transmission blocked |
| pantothenate kinase 1 | *pank1* | PANK1 | PBANKA_1022600 | Yes | No oocysts or sporozoites, transmission blocked |
| pantothenate kinase 2 | *pank2* | PANK2 | PBANKA_061140 | Yes | No oocysts or sporozoites, transmission blocked |
|  | *pank1 & 2* | PANK1 & 2 |  | Yes | No oocysts or sporozoites, transmission blocked |
| putative GABA transporter | *trp* | TRP | PBANKA_030670 | No | - |
